# Yield stability and GxE interaction of green-seeded fenugreek across diverse environments using multivariate models

**DOI:** 10.3389/fpls.2026.1917204

**Published:** 2026-09-22

**Authors:** Ravindra Singh, Ram S. Meena, Sharda Choudhary, Ravi Y, Ram D. Meena, Narottam K. Meena, Dayanand, Pooja K. Shekhawat, Ramesh K. Dular, Basant Kachouli, Amar C. Shivran, Shailesh Marker, Pawan Kumar, Balu R. Choudhary, Birbal, Vijay S. Rathore, Yogesh Kanojia, Arjun Singh, Upvan Gupta, Arvind K. Verma

**Affiliations:** 1 Indian Council of Agricultural Research-National Research Centre on Seed Spices, Ajmer, Rajasthan, India; 2 Krishi Vigyan Kendra, Jhunjhunu, Rajasthan, India; 3 Sardarkrushinagar Dantiwada Agricultural University, Sardarkrushinagar, Gujarat, India; 4 Krishi Vigyan Kendra, Fatehpur Shekhawati, Rajasthan, India; 5 Kailash Nath Katju, College of Horticulture, Mandsaur, Madhya Pradesh, India; 6 Sri Karan Narendra Agriculture University, Jobner, Rajasthan, India; 7 ICAR-Central Institute for Arid Horticulture, Bikaner, Rajasthan, India; 8 ICAR-Central Arid Zone Research Institute -Regional Research Station, Bikaner, Rajasthan, India; 9 Krishi Vigyan Kendra, Pratapgarh, Rajasthan, India; 10 Krishi Vigyan Kendra, Rajasthan, India; 11 Adaptive Trial Centre, Ajmer, Rajasthan, India; 12 Directorate of Floricultural Research, Pune, Maharashtra, India

**Keywords:** AMMI, fenugreek, GGE biplot, GxE interaction, MTSI, Which-Won-Where

## Abstract

Green-seeded fenugreek (*Trigonella foenum-graecum* L.) constitutes a valuable genetic resource owing to its elevated chlorophyll concentration and enhanced nutraceutical potential. However, information regarding its performance and stability across diverse agro-climatic conditions remains limited. The present study evaluated four green-seeded genotypes (IC-0633364, IC-0633365, IC-0633366, and IC-0633369) together with three commercial cultivars (Ajmer Fenugreek-4, Ajmer Fenugreek-5, and Hisar Manohar) across nine contrasting environments to elucidate genotype × environment (G×E) interactions and identify stable, high-performing genotypes. Eleven phenological, growth, and yield-related traits and eight biochemical attributes were assessed using combined analysis of variance, additive main effects and multiplicative interaction (AMMI), genotype plus genotype × environment (GGE) biplot, factor analysis, multi-trait stability index (MTSI) and Multi-Trait Genotype-Ideotype Distance Index (MGIDI). Combined ANOVA revealed highly significant (p < 0.001) effects of genotype, environment, and G×E interaction for most traits, highlighting substantial variation in genotype responses across environments. AMMI and GGE biplot analyses demonstrated distinct patterns of adaptation and trait expression, with AFg5 exhibiting superior performance for seed yield, biological yield, and pods per plant, whereas AFg4 excelled for plant height and test weight. Among the evaluated entries, IC 66, IC 65, and IC 69 consistently exhibited superior multi-trait performance and recorded the lowest MTSI values, indicating better multi-trait stability across environments; MGIDI analysis further supported the favourable multi-trait profiles of these genotypes based on their strength and weakness contributions. Furthermore, IC 65 possessed the highest chlorophyll content, while IC 66 exhibited superior protein and nitrogen concentrations. The integration of stability and multi-trait analyses demonstrated that green-seeded fenugreek can successfully combine agronomic resilience with enhanced biochemical quality. These genotypes constitute valuable breeding resources for the development of nutritionally superior and widely adapted fenugreek cultivars for diverse production environments.

## 1 Introduction

Fenugreek (*Trigonella foenum-graecum* L.) is an important seed spice crop belonging to the family Fabaceae and is widely cultivated across tropical and subtropical regions of the world. It is commercially grown in countries such as India, Pakistan, Iran, Egypt, Turkey, and several Middle Eastern and North African regions for its nutritional, medicinal, and industrial importance (Altuntaş et al., 2005). Fenugreek is a high-value legume crop known for its potential to improve human, animal, and soil health (Choudhary et al., 2024). Fenugreek seeds are valued for its nutritional and medicinal properties and represents an important source of chlorophyll, 4-hydroxyisoleucine (4-HIL), free amino acids, essential minerals, and vitamins (Singh et al., 2025a; Najafi et al., 2013; Al-Jasass and Al-Jasser, 2012; Leela and Shafeekh, 2008). Its seeds are small, hard, smooth in texture, and yellow to brownish-yellow in colour (Altuntaş et al., 2005). Although yellow- to brown-seeded types predominate in cultivated fenugreek, substantial variation in seed colour exists within the species. Naturally occurring green-seeded genotypes represent a distinct and relatively less-exploited component of fenugreek germplasm and have attracted increasing interest because of their characteristic green pigmentation and associated biochemical composition (Singh et al., 2025c; Singh and Choudhary, 2026). Reports of blue- and white-seeded phenotypes further indicate considerable genetic diversity for seed colour, which may involve one or a few major genetic factors, although the genetic basis of seed colour expression and its environmental modulation remain insufficiently understood (Ayvazyan et al., 2023; Bekele et al., 2020; Laxmi et al., 1983). Compared with conventional yellow-seeded types, some green-seeded genotypes have been reported to contain greater amounts of chlorophyll and certain nutritionally relevant compounds, including 4-HIL, free amino acids, phenolics, and antioxidant constituents (Singh et al., 2025a, Singh et al., 2025b). However, these observations should not be interpreted as evidence of universal biochemical superiority, because the accumulation of such compounds may vary with genotype, environment, developmental stage, and growing conditions (Singh et al., 2025a, Singh et al., 2025b). Among these, 4-HIL has received particular attention because of its role in improving insulin sensitivity and glucose metabolism (Broca et al., 2000; Ravikumar and Anuradha, 1999), highlighting the potential of green-seeded fenugreek for functional food and nutraceutical applications.

Fenugreek is predominantly cultivated during the rabi season under semi-arid and arid environments and is well adapted to well-drained loam soils with slightly alkaline pH (Acharya et al., 2008; Petropoulos, 2002). The development of stable and high-yielding varieties largely depends on the evaluation of genotypes under diverse agro-climatic conditions through multi-location trials (MLTs) (Yadav et al., 2020; Kena and Latera, 2025). In recent years, fenugreek cultivation has expanded beyond its traditional production areas owing to its wide-ranging culinary, nutritional, and medicinal importance (Güzel and Özyazıcı, 2021). However, substantial variation in cultivar performance across different agro-climatic regions has emerged as a major constraint to achieving stable productivity, with yields often differing markedly under diverse environmental conditions. Accordingly, comprehensive multi-environment evaluation of fenugreek germplasm is essential for identifying genotypes with superior adaptability, yield stability, and sustainability. This requirement is particularly relevant for green-seeded fenugreek because environmental variation may influence not only yield and phenological performance but also the expression or accumulation of quality-related biochemical traits. A genotype showing desirable seed colour or biochemical characteristics in one environment, may therefore not necessarily maintain these attributes across contrasting environments. Recent advances in integrated omics approaches have further emphasized the importance of exploiting genetic and biochemical variability in fenugreek for improvement of yield stability, nutraceutical quality, and stress resilience (Kachhwaha et al., 2024). Consequently, understanding genotype × environment (G×E) interactions through multi-environment trials is essential for identifying broadly adapted and stable genotypes suitable for diverse agro-ecological conditions. Despite increasing interest in the biochemical characteristics of green-seeded fenugreek, systematic multi-environment assessment integrating agronomic and biochemical performance remains limited. Therefore, evaluating the consistency of desirable agronomic and biochemical traits across contrasting environments is essential for determining the breeding potential of green-seeded fenugreek germplasm. Advanced statistical approaches such as additive main effects and multiplicative interaction (AMMI), genotype plus genotype × environment interaction (GGE) biplot and the Multi-Trait Stability Index (MTSI) provide powerful tools for dissecting genotype × environment interactions and identifying stable, high-performing genotypes (Yan and Kang, 2003; Olivoto et al., 2019). Therefore, the present study aimed to (i) evaluate the agronomic and biochemical performance of green-seeded fenugreek genotypes across contrasting environments, (ii) quantify genotype × environment interactions for yield and biochemical traits, (iii) identify stable, high-performing, and widely adapted genotypes and (iv) characterize representative and discriminating test environments for future fenugreek breeding and cultivar evaluation programmes.

## 2 Materials and methods

### 2.1 Experimental site and design

The study was conducted during the rabi season across nine contrasting environments located in Rajasthan and Madhya Pradesh, India, to evaluate genotype × environment (G×E) interactions, adaptability, and stability of fenugreek (*Trigonella foenum-graecum* L.) genotypes. The test environments included ICAR-NRCSS, Ajmer (E1); KVK Jhunjhunu (E2); KVK Maulasar, Nagaur (E3); K.N. K. College of Horticulture, Mandsaur (E4); ICAR-CIAH, Bikaner (E5); SKNAU, Jobner (E6); KVK Fatehpur, Sikar (E7); KVK Pratapgarh (E8); and Adaptive Trial Centre (ATC), Ajmer (E9). Details of the experimental sites, including geographical coordinates and altitude, are provided in **Supplementary Table S1**, and their spatial distribution is shown in **Figure 1**. The experiment was laid out in a randomized complete block design (RCBD) with four replications at each location. Each plot measured 3.0 × 3.0 m, with a crop geometry of 30 × 10 cm (row × plant spacing), resulting in 28 experimental plots at each location. The crop was fertilized with the recommended dose of 20:40:30 kg N:P:K ha^-^¹, and 5–6 surface irrigations were provided during the crop-growing period. Sowing dates and days to maturity for each location and growing season are presented in **Supplementary Table S24**.

**Figure 1:**
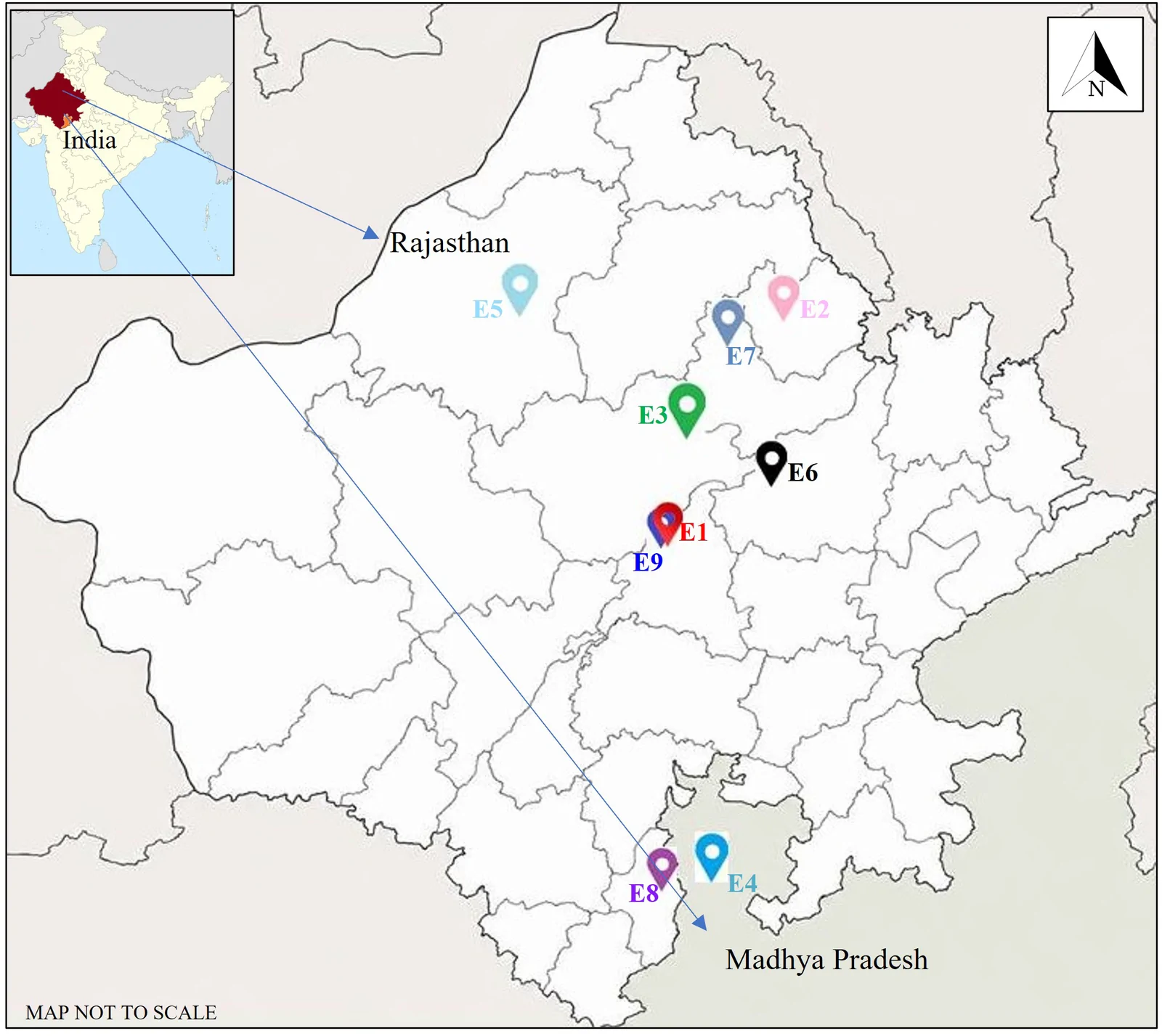
Geographical distribution of the nine experimental environments used for multi-environment evaluation of fenugreek genotypes across Rajasthan and Madhya Pradesh, India. The map shows the location, code, latitude, and longitude of each testing site: ICAR-NRCCS Ajmer (E1: 26.36° N, 74.59° E), KVK Jhunjhunu (E2: 28.10° N, 75.34° E), KVK Maulasar (E3: 27.31° N, 74.75° E), COH Mandsaur (E4: 24.05° N, 75.10° E), ICAR-CIAH Bikaner (E5: 28.01° N, 73.34° E), SKNAU Jobner (E6: 26.97° N, 75.37° E), KVK Fatehpur (E7: 28.01° N, 74.96° E), KVK Pratapgarh (E8: 24.02° N, 74.81° E), and ATC Ajmer (E9: 26.36° N, 74.59° E).

### 2.2 Meteorological observation

Meteorological data for each experimental location were obtained from the nearest meteorological observatories or agro-meteorological stations during the crop growing seasons (October-March) of 2023–24 and 2024-25. The recorded parameters included total rainfall (mm), minimum and maximum temperature (°C), and minimum and maximum relative humidity (%). Considerable variation in climatic conditions was observed among the test environments (**Supplementary Table S2**), providing a diverse range of agro-climatic conditions for evaluating genotype performance and stability. During the study period, seasonal rainfall ranged from 0.22 to 40.3 mm in 2023–24 and from 5.7 to 38.2 mm in 2024-25. Mean minimum and maximum temperatures varied from 3.3 to 16.5 °C and 26.0 to 38.0 °C, respectively, while relative humidity ranged from 20.1 to 59.9% (minimum) and 69.7 to 95.1% (maximum) across locations.

### 2.3 Soil analysis

Prior to sowing, representative soil samples (0–15 cm depth) were collected from each experimental site and analyzed using standard laboratory procedures to determine physicochemical properties. Soil organic carbon (OC), available nitrogen (N), phosphorus (P), potassium (K), pH, and electrical conductivity (EC) were determined, while irrigation water samples were analyzed for pH and EC (Choudhary et al., 2025). The experimental locations exhibited substantial variation in soil fertility and physicochemical characteristics. Organic carbon content ranged from 0.12 to 0.41%, available nitrogen from 119.3 to 265.2 kg ha**^-1^**, available phosphorus from 13.6 to 30.5 kg ha**^-1^**, and available potassium from 155.0 to 296.5 kg ha**^-1^**. Soil pH varied from 7.5 to 9.3, indicating neutral to moderately alkaline soil conditions, whereas soil EC ranged from 0.13 to 0.51 dS m**^-1^**. Irrigation water pH ranged between 7.6 and 8.1, with EC values varying from 0.76 to 3.04 dS m**^-1^** (**Supplementary Table S3**).

### 2.4 Plant material

The experimental material comprised seven fenugreek genotypes, including four advanced green-seeded entries [IC-0633364 (IC 64), IC-0633365 (IC 65), IC-0633366 (IC 66), and IC-0633369 (IC 69)] and three released cultivars used as checks [Ajmer Fenugreek-4 (AFg4), Ajmer Fenugreek-5 (AFg5), and Hisar Manohar (HM)]. The green-seeded entries were selected from a diverse fenugreek germplasm collection maintained at ICAR-National Research Centre on Seed Spices (ICAR-NRCSS), Ajmer. These lines were evaluated over four consecutive years for agronomic performance, seed colour stability, uniformity, and distinctiveness. Based on their consistent expression of green seed colour, superior performance, and phenotypic stability, four promising lines were identified and advanced for multi-environment evaluation. Details of the genotypes included in the study are presented in **Table 1**.

**Table 1:** Mean performance of locations and fenugreek genotypes for phenological, growth, and yield traits across multi-environment trials along with ANOVA parameters.

Environment (E)	ID	Trait*										
		DF	DM	PH	BPP	PPP	PL	SPP	SY	BY	TW	HI
ICAR-NRCSS Ajmer	E1	49.96	129.3	76.0	6.30	58.14	11.19	18.00	2359	11225	14.29	21.29
KVK Jhunjhunu	E2	51.46	126.3	72.4	6.26	42.18	10.88	17.73	1206	4873	12.31	24.73
KVK Maulasar	E3	50.55	122.4	69.5	7.37	50.38	9.27	16.59	966	3659	11.95	26.75
COH, Mandsaur	E4	54.52	110.7	79.9	5.52	44.52	10.93	18.74	1192	4102	11.42	27.65
CIAH Bikaner	E5	59.34	133.1	70.9	6.60	32.98	8.54	16.03	1663	5931	12.03	28.78
SKNAU Jobner	E6	54.50	128.5	70.4	5.70	36.58	9.87	15.55	1025	3819	14.19	27.05
KVK Fatehpur	E7	51.88	128.5	49.0	5.05	33.04	8.49	14.32	928	3694	11.59	25.58
KVK Pratapgarh	E8	56.50	128.0	79.1	6.07	64.50	11.76	19.34	2860	11214	12.95	25.57
ATC Ajmer	E9	49.07	128.7	74.7	6.56	73.59	12.39	19.39	2951	14778	14.05	20.32
SE (m)		0.17	0.2	1.2	0.12	1.10	0.11	0.22	37	172	0.08	0.36
C.D.		0.47	0.6	3.3	0.34	3.07	0.32	0.62	102	480	0.22	1.01
Genotype (G)												
IC-0633364	IC 64	52.94	127.6	67.7	5.99	46.66	10.53	17.17	1633	7276	12.91	24.17
IC-0633365	IC 65	53.36	125.3	70.1	6.38	46.44	10.29	17.38	1644	6583	12.59	26.10
IC-0633366	IC 66	53.36	125.9	70.0	6.16	48.16	10.17	17.51	1643	6643	12.52	25.24
IC-0633369	IC 69	54.24	126.7	71.3	6.43	46.16	10.18	17.34	1742	7220	12.98	26.05
Ajmer Fenugreek-4	AFg4	53.19	124.5	75.9	6.06	49.93	10.50	16.96	1746	7542	13.29	24.61
Ajmer Fenugreek-5	AFg5	51.16	124.7	74.8	6.03	51.24	10.55	16.98	1834	7688	13.08	25.26
Hisar Manohar	HM	53.35	128.4	69.3	6.08	50.44	10.35	17.75	1540	6276	11.90	25.69
SE (m)		0.15	0.2	1.0	0.11	0.97	0.10	0.20	32	152	0.07	0.32
C.D.		0.41	0.5	2.9	0.30	2.71	0.28	N/A	90	423	0.19	0.89
Interaction E*G												
SE (m)		0.45	0.6	3.1	0.32	2.91	0.30	0.59	97	455	0.21	0.96
C.D.		1.24	1.6	8.6	0.90	8.12	0.83	N/A	270	1270	0.58	2.68

*Trait abbreviations: DF, days to flowering; DM, days to maturity; PH, plant height cm; BPP, branches plant^-1^; PPP, pods plant^-1^; PL, pod length cm; SP, seeds pod^-1^; SY, seed yield kg ha^-1^; BY, biological yield kg ha^-1^; TW, test weight g; HI, harvest index

### 2.5 Growth and yield attributes

Eleven agronomic traits were evaluated, including days to flowering (DF), days to maturity (DM), plant height (PH), branches plant**^-^**^1^ (BPP), pods plant**^-^**^1^ (PPP), pod length (PL), seeds pod**^-^**^1^ (SPP), seed yield (SY), biological yield (BY), test weight (TW) and harvest index (HI). Observations on PH, BPP, PPP, PL, and SPP were recorded from five randomly selected representative plants in each plot. Seed yield and biological yield were recorded on a plot basis at harvest and expressed as kg ha**^-^**^1^. HI was calculated as the ratio of SY to BY and expressed as a percentage, whereas TW was determined as the weight of 1000 seeds (g) (**Table 1**).

### 2.6 Estimation of biochemical compounds

Fully matured seeds of green and yellow seeded fenugreek genotypes were harvested and analyzed for biochemical constituents (**Table 2**). Seed samples were collected separately from the four field replications (R1-R4) for each genotype × location combination, and the mean of the four replicate observations was used for subsequent statistical analyses. Total chlorophyll content (TCC) was estimated following the spectrophotometric method of Mazza and Oomah (1994). Ground seed samples (1g) were extracted using acetone and NH_4_OH solution, centrifuged at 5000 rpm, and absorbance was recorded at 663, 645, 626, and 700 nm using a UV-Vis spectrophotometer. Chlorophyll a and chlorophyll b were calculated separately and summed to obtain total chlorophyll content.

**Table 2:** Mean performance of locations and fenugreek genotypes for biochemical and nutritional traits across multi-environment trials along with ANOVA parameters.

Environment (E)	ID	Trait*							
		TCC (mg/g)	4-HIL (mg/gm)	DC (mg/100gm)	FAA (mg/gm)	P (mg/gm)	NC %	PC %	TSS (mg/gm)
NRCSS Ajmer	E1	3.26	11.46	319.2	14.55	0.96	4.03	25.19	2.68
KVK Jhunjhunu	E2	2.22	10.30	189.3	12.87	1.20	4.43	27.67	2.06
KVK Maulasar	E3	1.88	8.73	154.6	10.91	1.07	4.70	29.37	2.41
COH, Mandsaur	E4	2.27	7.43	207.5	9.29	1.05	4.11	25.68	2.10
CIAH Bikaner	E5	3.82	11.43	188.8	14.29	1.19	4.68	29.23	3.39
SKNAU Jobner	E6	1.31	8.66	186.0	10.82	1.21	4.58	28.64	1.46
KVK Fatehpur	E7	1.94	10.82	173.4	13.57	1.11	4.46	27.88	3.14
KVK Pratapgarh	E8	2.81	7.21	152.5	9.01	1.12	4.10	25.62	1.76
ATC Ajmer	E9	3.95	8.99	159.9	11.24	0.94	4.14	25.89	2.28
SE (m)		0.10	0.12	2.6	0.15	0.01	0.04	0.28	0.04
C.D.		0.29	0.33	7.4	0.42	0.03	0.12	0.77	0.10
Genotype (G)									
IC-0633364	IC 64	3.05	10.11	177.7	12.64	1.10	4.44	27.78	2.37
IC-0633365	IC 65	3.91	9.79	190.3	12.23	1.02	4.42	27.64	2.37
IC-0633366	IC 66	3.06	9.86	192.0	12.32	1.17	4.51	28.16	2.39
IC-0633369	IC 69	3.09	9.67	200.2	11.62	1.18	4.49	28.08	2.53
Ajmer Fenugreek-4	AFg4	1.30	8.48	191.2	10.60	0.98	4.35	27.19	2.48
Ajmer Fenugreek-5	AFg5	1.01	8.61	192.7	10.76	1.09	4.28	26.75	2.47
Hisar Manohar	HM	2.83	9.61	202.5	12.70	1.12	4.01	25.09	1.94
SE (m)		0.09	0.10	2.3	0.13	0.01	0.04	0.24	0.03
C.D.		0.25	0.29	6.5	0.37	0.03	0.11	0.68	0.09
Interaction E*G									
SE (m)		0.27	0.31	7.0	0.40	0.03	0.12	0.73	0.09
C.D.		0.75	0.87	19.5	1.10	0.07	0.33	2.05	0.26

*Trait abbreviations: TCC, Total chlorophyll content; 4-HIL, 4-hydroxyisoleucine; DC, Diosgenin content; FAA, Free Amino Acids; P, Phenol; NC, Nitrogen content; PC, Protein Content; TSS, Total soluble sugars.

The 4-hydroxyisoleucine (4-HIL) content was estimated using the rapid screening method of Rathore et al. (2014). Seed samples were extracted with 80% ethanol, followed by ethyl acetate extraction and concentration by evaporation. Total free amino acids were quantified by the ninhydrin method using leucine as the standard, and 4-HIL was estimated as 80% of the total free amino acid content. For each genotype × environment combination, the analysis was performed in four analytical replicates, and their mean value was used for statistical analysis. Diosgenin content (DC) was determined after ethanol extraction of seed powder with continuous shaking for 48 h. The extract was centrifuged, concentrated, and reacted with p-anisaldehyde and sulfuric acid reagents for color development. Absorbance was measured at 430 nm and diosgenin concentration was quantified using a standard calibration curve prepared in ethyl acetate.

Free amino acids (FAA) were extracted with 80% ethanol and quantified by the ninhydrin method following Lee and Takahashi (1966). Nitrogen content (NC) in seed was estimated by the Kjeldahl method and protein content (PC) was calculated by multiplying nitrogen content with a factor of 6.25 (AOAC, 2000). Total soluble sugars (TSS) were extracted in 80% ethanol and estimated by the anthrone method, with absorbance measured at 600 nm using glucose as standard (Francis et al., 1971).

### 2.7 Statistical analysis

All statistical analyses were performed using R v4.3.2 via RStudio, with the metan package (Olivoto and Lúcio, 2020). The analyses included combined analysis of variance (ANOVA), Additive Main Effects and Multiplicative Interaction (AMMI) analysis, Genotype plus Genotype × Environment Interaction (GGE) biplot analysis, factor analysis, estimation of broad-sense heritability and selection gain, and Multi-Trait Stability Index (MTSI) and MGIDI analysis. Data from the two growing seasons (2023–24 and 2024-25) were averaged within each location to obtain nine location-based test environments (E1-E9), which were subsequently used for the AMMI, GGE biplot, MTSI and MGIDI analyses. Meteorological observations for both growing seasons are provided in the **Supplementary Material** (**Supplementary Table S2**) to characterize the climatic conditions prevailing at the test locations and provide environmental context for interpreting genotype performance; these observations were not treated as additional test environments in the statistical analyses.

#### 2.7.1 Analysis of variance

A combined analysis of variance (ANOVA) across environments was performed to assess the effects of genotype (G), environment (E), and genotype × environment interaction (G×E) on phenological, agronomic, yield, and biochemical traits. The pooled ANOVA partitioned the total variation into its respective sources and tested their significance.

The statistical model used was:


$${\text{Y}}_{\text{ijk}}=\text{μ}+{\text{E}}_{\text{i}}+\text{R}{\left(\text{E}\right)}_{\text{ij}}+{\text{G}}_{\text{k}}+{\left(\text{GE}\right)}_{\text{ik}}+{\text{ϵ}}_{\text{ijk}}$$

where Y_ijk_ is the observed value of the k^th^ genotype in the j^th^ replication within the i^th^ environment; μ is the overall mean; G_k_ is the effect of the k^th^ genotype; E_i_ is the effect of the i^th^ environment; (GE)*_ik_* is the genotype × environment interaction effect; R(E)*ij* is the effect of the j^th^ replication nested within i^th^ environment and ϵ_ijk_ is the random residual error associated with the observation (Crossa, 1990).

The significance of genotype, environment, and G×E interaction effects was tested using appropriate F-tests. Significant G×E interactions indicated differential genotype responses across environments and justified the application of stability analyses.

#### 2.7.2 AMMI and GGE biplot analyses

AMMI (Additive Main Effects and Multiplicative Interaction) and GGE biplot (Genotype plus Genotype × Environment) analysis were performed to identify winning genotypes, mega-environments, and genotype × environment interaction patterns for yield-related and biochemical traits.

The AMMI model was expressed as:


$${\text{Y}}_{ij}=\text{μ}+{\text{G}}_{i}+{\text{E}}_{j}+\Sigma \;{\text{λ}}_{\text{n}}{\text{α}}_{\text{in}}{\text{γ}}_{\text{jn}}+{\text{ρ}}_{ij}$$

where Y*_ij_* is the mean performance of the *i* genotype in the *j* environment; μ is the grand mean; G*_i_* and E_j_ are the genotype and environment main effects, respectively; λ_n_ is the singular value of the n^th^ interaction principal component axis (IPCA); α_in_ and γ_jn_ are the genotype and environment eigenvector scores corresponding to the n^th^ IPCA; and ρij is the residual associated with genotype *i* in environment *j*.

The GGE biplot was generated through singular value decomposition (SVD) of environment-cantered data by using IPCA I and II (principal components) and was used to visualize genotype performance, stability, genotype ranking, environment ranking, and “which-won-where” patterns across environments (Yan and Kang, 2003).

The GGE model was:


$${\text{Y}}_{ij}-\text{μ}-{\text{β}}_{j}={\text{λ}}_{1}{\text{ξ}}_{\text{i}1}{\text{η}}_{\text{j}1}+{\text{λ}}_{2}{\text{ξ}}_{\text{i}2}{\text{η}}_{\text{j}2}+{\text{ϵ}}_{ij}$$

where β*_j_*is the main effect of the j^th^ environment; λ_1_ and λ_2_ are the singular values associated with the first and second principal components (PC1 and PC2), respectively; ξ*_i_*_1_ and ξ*_i_*_2_ are the genotype scores for PC1 and PC2; ηj1 and ηj2 are the environment scores for PC1 and PC2; and ϵ*_ij_* is the residual associated with genotype *i* in environment *j*.

The relative contributions of genotype (G), environment (E), and genotype × environment interaction (G×E) to the combined G + E + G×E variation were calculated from the corresponding sums of squares (SS), obtained as the product of mean square (MS) and degrees of freedom (*df*):


SS=Mean Square x df

The relative contribution of genotype, environment and G×E was calculated as:


$$\text{G }(\%)=\frac{{\text{SS}}_{\text{G}}}{{\text{SS}}_{\text{G}}+{\text{SS}}_{\text{E}}+{\text{SS}}_{\text{G x E}}}\times 100$$

$$\text{E }(\%)=\frac{{\text{SS}}_{\text{E}}}{{\text{SS}}_{\text{G}}+{\text{SS}}_{\text{E}}+{\text{SS}}_{\text{G x E}}}\times 100$$

$$\text{G x E }(\%)=\frac{{\text{SS}}_{\text{G x E}}}{{\text{SS}}_{\text{G}}+{\text{SS}}_{\text{E}}+{\text{SS}}_{\text{G x E}}}\times 100$$

The contribution of each AMMI interaction principal component was calculated as:


$${\text{IPCA}}_{\text{k}}\;(\%)=\frac{{\text{SS}}_{\text{IPCAk}}}{{\text{SS}}_{\text{G x E}}}\times 100$$

where k=1 or 2 for IPCA1 and IPCA2, respectively. The significance of G×E interaction and individual IPCA was assessed using the corresponding F-tests at *P* ≤ 0.05, *P* ≤ 0.01, and *P* ≤ 0.001. The mean square estimates are presented in **Table 3A**, while the relative contributions of G, E, and G×E and the proportions of G×E interaction accounted for by IPCA1 and IPCA2 are presented in **Table 3B**.

Table 3Mean square estimates of AMMI ANOVA and relative contributions of genotype, environment, and genotype × environment interaction for 17 phenological, growth, yield, and biochemical traits of seven fenugreek genotypes across multi-environment trials.

**Table:** 

A. Mean square estimates of AMMI ANOVA																		
Source of variation	*df*	DF	DM	PH	BPP	PPP	PL	SPP	SY	BY	TW	HI	TCC	4-HIL	DC	P	NC	TSS
Environment (E)	8	273.16***	1159.49***	2353.08***	12.88***	5811.93***	54.74***	88.38***	19031299***	498000000***	41.59***	224.27***	23.28***	72.53***	72846***	0.27***	1.98***	10.88***
Replication	27	1.89	1.61	93.52***	0.99***	45.33	0.48	3.02***	55081*	565000	0.28**	1.89	0.43	0.54	217	0.003	0.25***	0.04
Genotype (G)	6	46.02***	77.17***	322.10***	1.15*	170.70***	0.95*	2.93*	336671***	10300000***	4.69***	18.69***	39.93***	14.47***	2317***	0.19***	1.06***	1.38***
(E x G)	48	43.25***	19.04***	118.10***	0.62*	146.63***	0.92***	1.34	130636***	3960000***	2.08***	5.89*	1.91***	2.14***	2600***	0.02***	0.08***	0.60***
IPCA 1	13	138.76***	53.32***	263.62***	1.32***	262.16***	2.03***	2.83**	216453***	10700000***	4.76***	9.22**	6.37***	3.91***	6439***	0.03***	0.13***	1.36***
IPCA 2	11	12.49***	10.86***	126.78***	0.55	181.69***	0.58	1.37	190828***	2320000**	2.11***	8.97*	0.51	2.15***	1600***	0.02***	0.10**	0.52***
Residual	24	5.62	4.22	35.29	0.28	67.98	0.47	0.53	56562.75	1040000	0.62	2.68	0.13	1.18	978.46	0.01	0.04	0.23
Error	162	1.76	1.38	31.23	0.4	33.29	0.35	1.22	35225	866000	0.15	3.94	0.29	0.35	188	0.003	0.04	0.03
Total	251	23.24	39.58	132.7	0.87	228.13	2.01	3.79	581957	15300000	1.98	10.57	2.24	3.35	2952	0.02	0.14	0.53
B. Relative contribution of G, E and G × E and AMMI interaction partitioning.																		
Trait	G (%)			E (%)			G×E (%)			IPCA1 (% G×E)			IPCA2 (% G×E)			G×E significance		
DF	6.09			48.16			45.75			86.89			6.62			***		
DM	4.35			87.07			8.58			75.84			13.07			***		
PH	7.31			71.24			21.45			60.45			24.60			***		
BPP	4.94			73.76			21.30			57.66			20.33			*		
PPP	1.88			85.22			12.90			48.42			28.40			***		
PL	1.17			89.78			9.05			59.76			14.45			***		
SPP	2.23			89.62			8.15			57.20			23.43			NS		
SY	1.26			94.84			3.91			44.87			33.48			***		
BY	1.46			94.05			4.49			73.18			13.43			***		
TW	6.11			72.22			21.67			61.98			23.25			***		
HI	5.12			81.96			12.92			42.40			34.90			*		
TCC	46.30			35.99			17.72			90.33			6.12			***		
4-HIL	11.28			75.38			13.34			49.48			23.02			***		
DC	1.93			80.78			17.30			67.07			14.10			***		
P	26.76			50.70			22.54			40.63			22.92			***		
NC	24.42			60.83			14.75			44.01			28.65			***		
TSS	6.67			70.13			23.20			61.39			19.86			***		

*df*, degree of freedom; DF, days to flowering; DM, days to maturity; PH, plant height; BPP, branches plant^-1^; PPP, pods plant^-1^; PL, pod length; SPP, seeds pod^-1^; SY, seed yield; BY, biological yield; TW, test weight; Hi, harvest index; TCC, Total chlorophyll content; 4-HIL, 4-hydroxyisoleucine; DC, Diosgenin content; P, Phenol; NC, Nitrogen content; TSS, Total soluble sugars.

*, **, and *** indicate significance at P ≤ 0.05, P ≤ 0.01, and P ≤ 0.001, respectively.

#### 2.7.3 Factor analysis, heritability and selection gain

Factor analysis was performed to identify the underlying structure of relationships among agronomic, yield, and biochemical traits. Factors with eigenvalues greater than one were retained and subsequently subjected to orthogonal varimax rotation (Kaiser, 1958). Rotated factor loadings were used to identify the major latent factors governing associations among traits and to determine their relative contributions to genotype differentiation.

Broad-sense heritability was estimated on a genotype-mean basis across locations using variance components derived from the combined ANOVA. The genotypic variance (σ²_G_), genotype × environment interaction variance (σ²_GE_), and residual error variance (σ²_E_) were estimated from the expected mean squares as:


$$\text{Genotypic variance}:\;\sigma_{\text{G}}^{2}=({\text{MS}}_{\text{G}}-{\text{MS}}_{\text{GxE}})/\text{er}$$

$$\text{G}\times \text{E variance}:{\text{ σ}}_{\text{GE}}^{2}=({\text{MS}}_{\text{GxE}}-{\text{MS}}_{\text{E}})/\text{r}$$

$$\text{Residual}/\text{error variance}:{\text{ σ}}_{\text{E}}^{2}={\text{MS}}_{\text{E}}$$

where (MS_G_) is the mean square for genotype, (MS_GE_) is the mean square for genotype × environment interaction, (MS_E_) is the residual mean square, (*e*) is the number of environments, and (*r*) is the number of replications. Heritability was then calculated as:


$$H^{2}=\frac{{\text{σ}}_{\text{G}}^{2}}{\left({\text{σ}}_{\text{G}}^{2}+\frac{{\text{σ}}_{\text{GE}}^{2}}{e}+\frac{{\text{σ}}_{\text{E}}^{2}}{er}\right)}$$

The expected selection gain (SG) was calculated as:


$$\text{SG }(\%)=[(\bar{\text{X}}\text{s}-\bar{\text{X}}\text{o})/\;\bar{\text{X}}\text{o}]\times {\text{H}}^{2}\times 100$$

where $\bar{\text{X}}\text{s}$ is the mean performance of the selected genotypes, $\bar{\text{X}}\text{o}$ is the overall population mean, H² is the broad-sense heritability, and SG represents the expected selection gain achieved through selection.

#### 2.7.4 Multi-trait stability index

The Multi-Trait Stability Index (MTSI) was computed to identify genotypes combining superior agronomic performance, biochemical quality, and environmental stability. Estimation of the index was based on the euclidean distance between genotype and ideotype scores obtained from factor analysis, following the procedure proposed by Olivoto et al. (2019).


$${\text{MTSI}}_{i}=\surd [\sum_{\text{j}=1}^{f}{\left(F_{ij}-F_{j}\right)}^{2}]$$

where F*_ij_* is the score of the *i*^th^ genotype for the *j*^th^ factor, F*_j_* is the corresponding score of the ideotype for the *j*^th^ factor, and f is the total number of retained factors. Lower MTSI values indicate genotypes that are closer to the ideal genotype and therefore more desirable.

#### 2.7.5 Multi-trait genotype-ideotype distance index

The Multi-Trait Genotype-Ideotype Distance Index (MGIDI) was estimated to identify genotypes with desirable overall performance across multiple agronomic and biochemical traits. The analysis was performed using the RAISINS web-based platform (Hisham et al., 2025), following the methodology proposed by Olivoto and Nardino (2021). MGIDI integrates the correlation structure among traits through factor analysis and quantifies the distance of each genotype from the predefined ideotype in the factor space. The ideotype was specified according to the desired direction of selection for each trait. The analysis was conducted using the R statistical environment (R Core Team, 2024).

## 3 Results

### 3.1 Mean performance

The pooled mean performance of genotypes and environments for phenological, growth, yield, and biochemical traits is presented in (**Tables 1**, **2**). Considerable variation was observed among the test environments for most traits, indicating substantial environmental diversity across the experimental locations. Among the environments, E9 recorded the highest mean SY (2951 kg ha^-1^), BY (14778 kg ha^-1^), PPP (73.59) and PL (12.39 cm), whereas E8 exhibited superior performance for PPP (64.50) and SPP (19.34). The highest TW was observed at E1 (14.29 g), while the lowest mean seed yield was recorded at E7 (928 kg ha^-1^), highlighting the pronounced influence of environmental conditions on trait expression.

Among the genotypes, AFg5 exhibited the highest mean SY (1834 kg ha^-1^) along with superior performance for PPP (51.24) and BY (7688 kg ha^-1^), whereas AFg4 recorded maximum PH (75.9 cm) and TW (13.29 g). Genotype IC 69 showed comparatively higher HI (26.05%), while HM produced the maximum number of SPP (17.75). The observed wide range of variation among genotypes for agronomic traits indicated the presence of substantial genetic variability, which is highly desirable for effective selection under multi-environment testing conditions.

Substantial environmental and genotypic variation was also observed for biochemical traits (**Table 2**), indicating strong differential expression of metabolite accumulation across environments. E9 and E5 recorded higher TCC (3.95 and 3.82 mg g^-1^ seed, respectively), while E1 exhibited the highest estimated 4-HIL content (11.46 mg g^-1^) and DC (319.2 mg 100 g^-1^). Among genotypes, IC 65 exhibited the highest TCC (3.91 mg g^-1^ seed), whereas HM recorded maximum DC (202.5 mg 100 g^-1^) and FAA (12.70 mg g^-1^). IC 66 recorded the highest protein (28.16%) and nitrogen (4.51%) contents, followed closely by IC 69. The pronounced variation observed for biochemical traits across environments further emphasized the importance of genotype × environment interactions and highlighted the potential for identifying stable green-seeded genotypes with superior biochemical attributes.

### 3.2 Analysis of variance

The analysis of variance for phenological, growth, yield, and biochemical traits is presented in **Supplementary Tables S4–S22**. Genotypic effects were highly significant (p < 0.001) for all evaluated traits, indicating the existence of substantial genetic variability among the fenugreek genotypes. Environmental effects were also highly significant (p < 0.001), demonstrating the strong influence of contrasting agro-climatic conditions on trait expression. Furthermore, significant genotype × environment (G × E) interactions were observed for most traits, indicating differential genotype responses across environments and the presence of considerable environmental modulation of trait performance. AMMI ANOVA further revealed significant genotype (G), environment (E), and genotype × environment (G × E) effects for most traits (**Tables 3A, B**). Environment was the major contributor to total G + E + G × E variation for most traits, accounting for 94.84% of the variation in seed yield (SY), 94.05% in biological yield (BY), 89.78% in pod length (PL), and 89.62% in seeds pod^-1^ (SPP). In contrast, genotype contributed most strongly to total chlorophyll content (TCC; 46.30%), followed by phenol (26.76%) and nitrogen content (NC; 24.42%). The contribution of G × E was highest for days to flowering (DF; 45.75%), followed by total soluble sugars (TSS; 23.20%), phenol (22.54%), test weight (TW; 21.67%), and plant height (PH; 21.45%). The G × E interaction was significant for 16 traits, whereas SPP showed a non-significant interaction. IPCA1 accounted for 40.63-90.33% of the G × E interaction sum of squares, while IPCA2 accounted for 6.12-34.90%. Together, IPCA1 and IPCA2 captured 63.54-96.44% of the G × E interaction across traits, indicating that the first two interaction principal components adequately represented the major patterns of G × E interaction.

### 3.3 G × E interaction analysis and selection of stable genotypes

#### 3.3.1 AMMI analysis

##### 3.3.1.1 AMMI I biplot analysis of genotype × environment interaction

The AMMI analysis for phenological, yield-attributing, and biochemical traits revealed significant genotype × environment (G × E) interactions across the evaluated environments (**Figure 2**; **Supplementary Figure S1**). AMMI1 biplots were generated using trait means on the x-axis and IPCA1 scores on the y-axis to assess genotype performance and G × E interaction. Genotypes combining higher mean performance with relatively smaller IPCA1 values were considered comparatively stable, whereas genotypes showing larger IPCA1 values exhibited greater environmental responsiveness.

**Figure 2:**
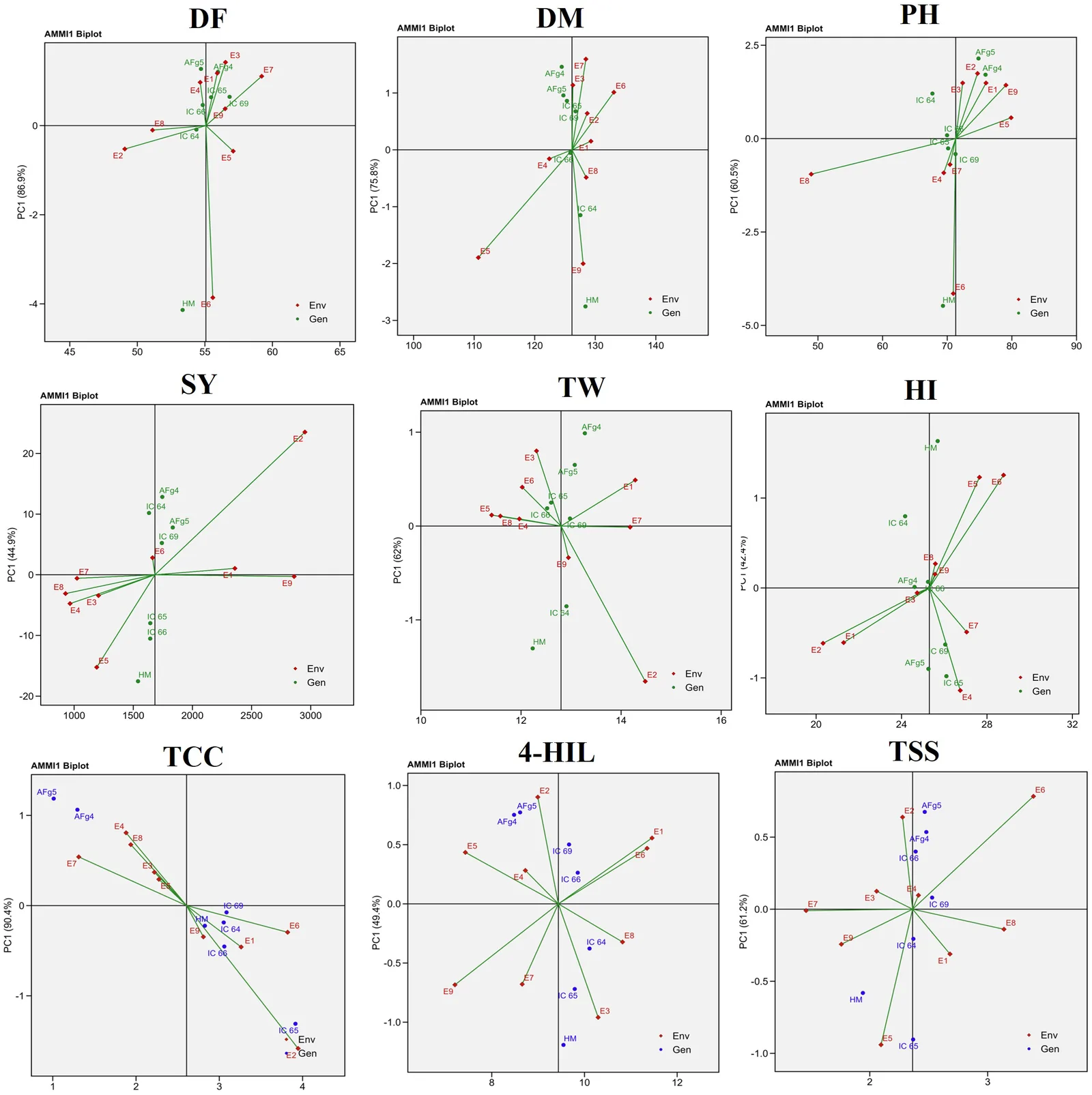
AMMI 1 biplots illustrating genotype × environment (G × E) interactions and stability patterns of seven fenugreek genotypes evaluated across nine environments for phenological, growth, and yield-attributing and biological traits. The biplots display the relationship between genotype and environmental responses based on trait means and Interaction Principal Component Axis 1 (IPCA1), enabling identification of stable and specifically adapted genotypes across environments.

For phenological traits, IC 64, IC 65, and AFg4 were positioned closer to the origin for days to flowering and maturity, indicating greater stability across environments. AFg4 exhibited higher mean performance for PH, whereas IC 69 showed relatively stable performance for branches plant^-1^. For yield-attributing traits, AFg5 and AFg4 displayed superior performance for pods plant^-1^ and pod length, particularly in association with productive environments such as E8 and E9, while HM exhibited specific adaptation for seeds pod^-1^.

For yield traits, AFg5, followed by AFg4 and IC 69, recorded higher mean seed yield and showed positive associations with productive environments, particularly E8 and E9, indicating specific adaptation to favourable growing conditions. Similarly, AFg5 and AFg4 exhibited superior performance for biological yield, whereas IC 65 and IC 69 demonstrated greater stability for test weight and harvest index, respectively.

Among the biochemical traits, IC 65 exhibited stable performance for total chlorophyll content, IC 64 exhibited higher mean performance and relatively stable expression for estimated 4-HIL content. HM displayed specific adaptation for diosgenin accumulation. IC 64 and HM were relatively stable for free amino acid content, IC 69 and IC 66 exhibited superior performance for phenol and protein content, respectively. IC 66 showed stable and superior expression of nitrogen content, while IC 69 was identified as the most stable genotype for total soluble sugar content.

Overall, the AMMI1 biplots differentiated genotypes according to their mean performance and IPCA1 scores, with AFg5 and AFg4 showing superior performance for several yield-related traits, whereas IC 64, IC 65, and IC 66 generally exhibited comparatively smaller interaction effects for several biochemical and quality-related traits.

##### 3.3.1.2 AMMI 2 biplot analysis of genotype × environment interaction

The AMMI2 biplot analysis provided further insight into the magnitude and pattern of genotype × environment (G × E) interactions by simultaneously considering IPCA1 and IPCA2 scores for phenological, yield-attributing, and biochemical traits across nine environments (**Figure 3**; **Supplementary Figure S2**). The AMMI2 biplot differentiated genotypes according to the magnitude and pattern of their G × E interaction. Genotypes located near the biplot origin contributed relatively less to G × E interaction, whereas those positioned farther from the origin showed stronger and more environment-specific responses. Their adaptation was interpreted in conjunction with their mean performance across environments.

**Figure 3:**
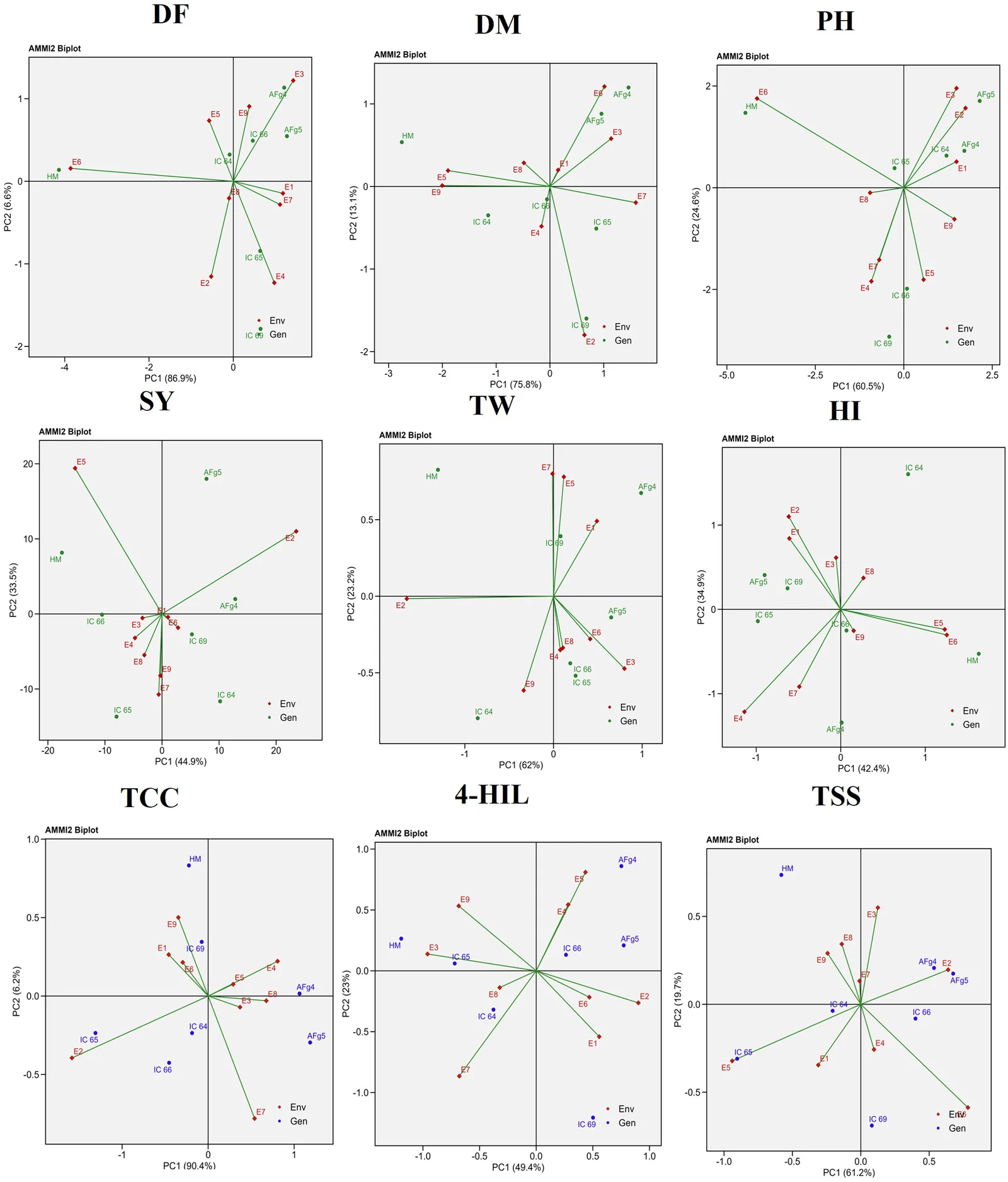
AMMI2 biplots depicting the magnitude and pattern of genotype × environment (G × E) interactions among seven fenugreek genotypes evaluated across nine environments for key phenological, growth, and yield-attributing and biochemical traits. The biplots were generated using the first two interaction principal component axes (IPCA1 and IPCA2) derived from AMMI analysis, enabling simultaneous assessment of genotype stability, environmental influence, and specific adaptation. Genotypes and environments located near the origin indicate broad adaptability and greater stability, whereas those positioned farther from the origin exhibit stronger interaction effects and specific environmental responsiveness.

For phenological traits, IC 64, IC 65, and AFg4 were positioned close to the biplot origin for days to flowering and days to maturity, indicating greater stability across environments. E5 and E1 showed greater interaction effects due to delayed flowering and maturity duration. AFg4 exhibited stronger interaction with environments E4 and E9 for plant height, whereas IC 69 showed specific adaptation for branches plant^-1^.

For yield-attributing traits, AFg5 and AFg4 exhibited stronger interactions for pods plant**^-1^** and pod length, particularly in association with productive environments E8 and E9, indicating specific adaptation to favourable conditions. HM exhibited specific adaptation for seeds pod^-1^). Seed yield showed pronounced crossover interaction, with AFg5, AFg4, and IC 69 showing strong associations with E9 and E8, which recorded the highest mean seed yields. Their high performance in these environments indicated greater responsiveness to favourable production conditions. In contrast, IC 64 and IC 65 showed relatively smaller interaction effects and more consistent performance across the tested environments.

For biological yield, AFg5 and AFg4 showed stronger associations with high-yielding environments E9 and E1, indicating specific adaptation for biological yield. Test weight exhibited comparatively stable responses for IC 65 and IC 69 across environments. Harvest index showed relatively lower interaction effects, although E4 displayed stronger environmental influence due to its higher harvest index values.

The biochemical traits also exhibited substantial G × E interactions. IC 65 showed stable performance for total chlorophyll content, while IC 64 exhibited favourable expression and specific adaptation for 4-hydroxyisoleucine (4-HIL) content. HM demonstrated positive interaction for diosgenin accumulation, particularly under E1 conditions. For free amino acids IC 64 and HM were positioned closer to the origin, indicating greater stability across environments. Phenol content exhibited pronounced environmental responsiveness, with IC 69 and IC 66 showing stronger interactions with environments E6 and E2. Protein content revealed specific adaptation of IC 66 to environments E3 and E5, corresponding to its higher mean protein accumulation. Nitrogen content exhibited similar interaction patterns, whereas IC 69 exhibited relatively stable performance and favourable adaptation for total soluble sugar.

Overall, the AMMI2 biplot provided a clearer representation of the magnitude and pattern of genotype × environment interactions, highlighting environment-specific associations and distinguishing genotypes with relatively smaller interaction effects from those showing stronger environmental responsiveness.

#### 3.3.2 Vector-view GGE biplots analysis of genotype × environment interaction

The vector-view GGE biplot analysis revealed substantial genotype and genotype × environment (G × E) interaction effects for phenological, yield-attributing, and biochemical traits across the nine test environments (**Figure 4**; **Supplementary Figure S3**). The vector-view GGE biplot illustrated genotype-environment relationships and helped identify genotypes showing favourable performance under particular environmental conditions. Genotype performance and environmental responsiveness were interpreted from the relative relationships among genotype and environment vectors, together with the observed mean performance. For phenological traits, IC 64 and IC 65 were located closer to the biplot origin for days to flowering and maturity, indicating greater stability across environments. AFg4 exhibited a longer vector and positive association with plant height, whereas IC 69 showed favourable adaptation for branches plant^-1^. For yield-attributing traits, AFg5 and AFg4 were strongly associated with pods plant**^-^**^1^ and pod length, particularly in productive environments E8 and E9, indicating specific adaptation to favourable growing conditions. HM exhibited specific adaptation for seeds pod^-^¹.

**Figure 4:**
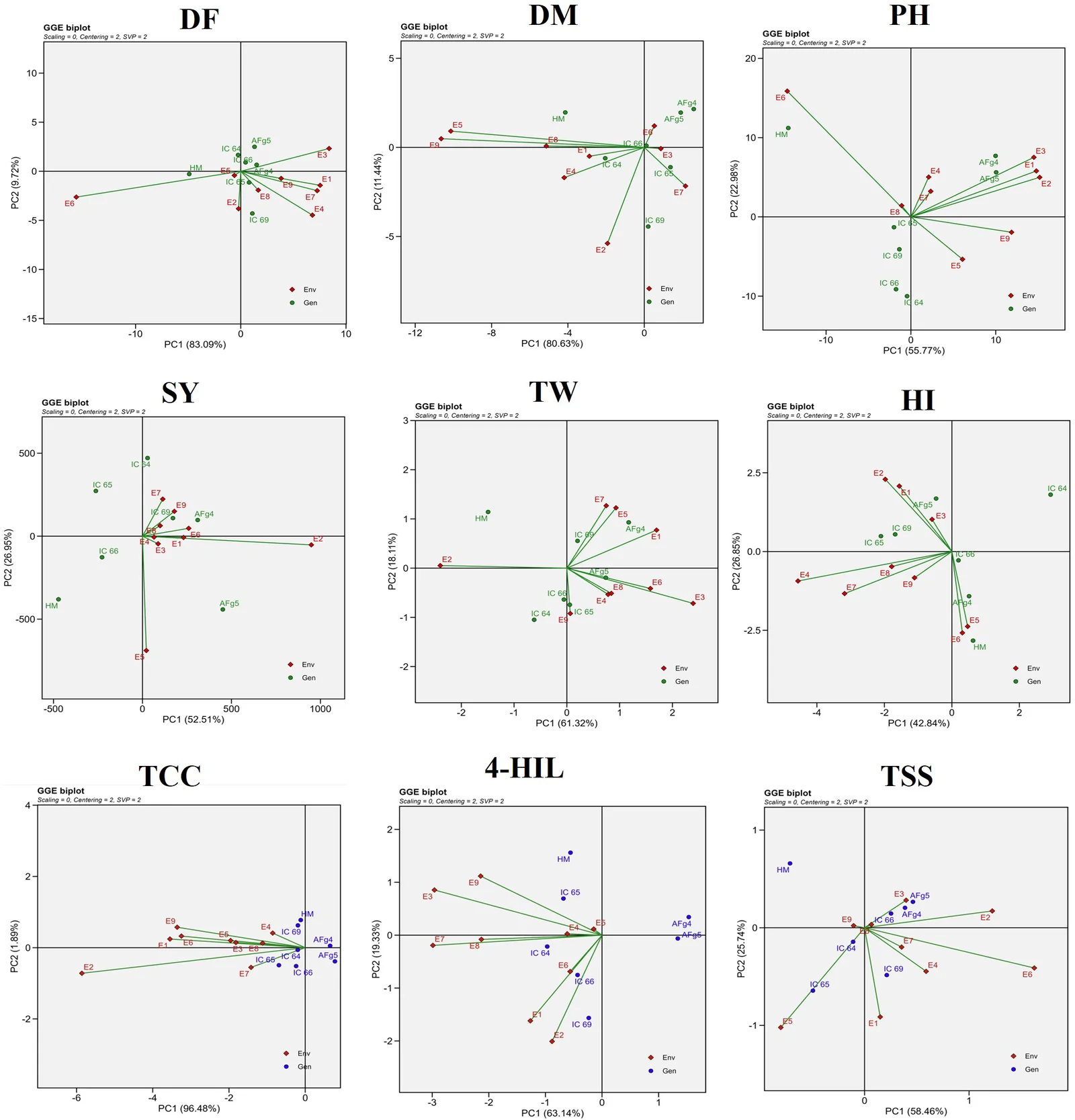
Vector-view GGE biplots depicting genotype main effects (G) and genotype × environment (G × E) interactions among seven fenugreek genotypes evaluated across nine environments for key phenological, growth, and yield-attributing and biochemical traits. The biplots illustrate the magnitude and direction of relationships among genotypes and environments, enabling visualization of trait-specific adaptation, stability, and interaction patterns based on the first two principal components (PC1 and PC2).

For yield traits, AFg5, followed by AFg4 and IC 69, showed superior performance and stronger associations with high-yielding environments for seed yield and biological yield. In contrast, IC 65 and IC 69 were positioned closer to the origin for test weight and harvest index, respectively, indicating greater stability across environments.

Among the biochemical traits, IC 65 exhibited stable performance for total chlorophyll content whereas IC 64 showed favourable expression and adaptation for 4-hydroxyisoleucine (4-HIL) content. IC 64 and HM exhibited favourable adaptation for free amino acid content, whereas HM showed specific adaptation for diosgenin accumulation. IC 66 and IC 69 exhibited favourable adaptation for phenol content, while IC 66 exhibited favourable adaptation and higher expression for protein and nitrogen contents. IC 69 was also identified as a stable genotype for total soluble sugar content.

Overall, the vector-view GGE biplot revealed differential genotype responses across environments. AFg5, AFg4, and IC 69 showed superior performance for yield-related traits, particularly under favourable environments, whereas IC 64, IC 65, IC 66, and HM showed favourable performance for several biochemical traits across the tested environments.

#### 3.3.3 Discriminativeness vs. representativeness (D/R biplot) of environments

The GGE biplot “discriminativeness versus representativeness” analysis revealed considerable variation among environments for phenological, yield-attributing, and biochemical traits (**Figure 5**; **Supplementary Figure S4**). Environments with longer vectors exhibited greater discriminating ability, whereas those with smaller angles to the average environment axis were considered more representative.

**Figure 5:**
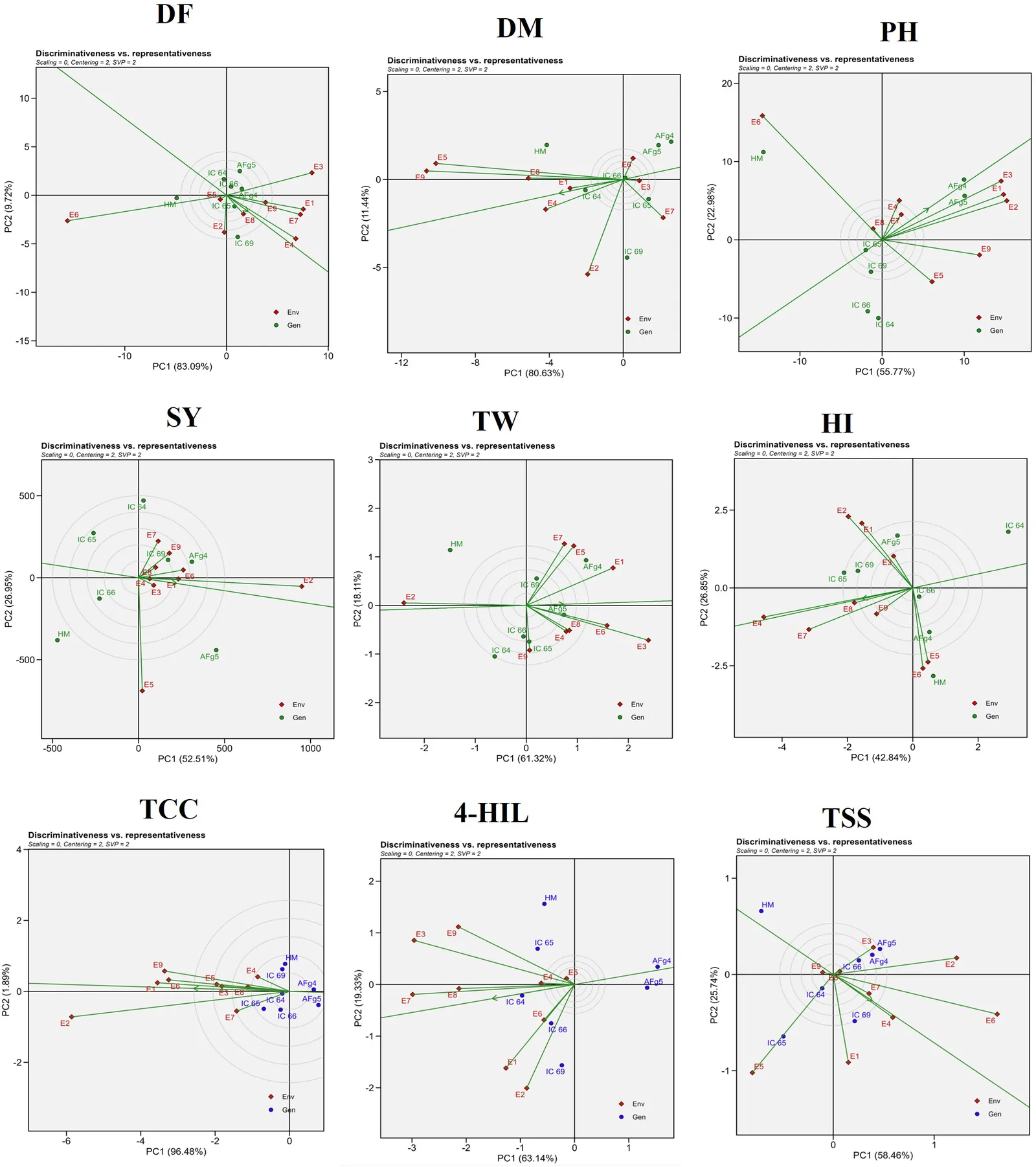
GGE biplot “Discriminativeness vs. Representativeness” views illustrating genotype performance and stability based on genotype main effects (G) and genotype × environment (G × E) interactions among seven fenugreek genotypes evaluated across nine environments. The concentric-circle biplots facilitate comparison of genotypes with the ideal genotype by simultaneously assessing mean performance, discriminating ability, and stability across environments.

For phenological and growth traits, E1 and E5 were more representative for flowering and maturity, whereas E9, E8, and E3 showed greater discriminating ability for growth and yield-attributing traits. For yield traits, E9 emerged as the most discriminating environment for seed yield and biological yield, followed by E8, while test weight and harvest index exhibited comparatively lower environmental discrimination.

Among biochemical traits, E1 and E5 were highly informative for chlorophyll, 4-hydroxyisoleucine (4-HIL), diosgenin, and free amino acid content, whereas E3 and E5 were more discriminating for protein and nitrogen contents. Overall, E9 and E8 were the most informative environments for evaluating yield-related traits, whereas E1 and E5 were more suitable for assessment of biochemical traits.

#### 3.3.4 Which -Won-Where model of GGE biplot

The “Which-Won-Where” GGE biplot revealed distinct mega-environment patterns and differential adaptation of fenugreek genotypes across environments for phenological, yield-attributing, and biochemical traits (**Figure 6**; **Supplementary Figure S5**). Vertex genotypes located at the polygon corners represented the highest-performing genotypes within their respective sectors, indicating genotype-specific superiority under the associated environmental conditions. Genotypes located near the origin showed relatively lower differentiation among environmental sectors and were not interpreted as broadly adapted solely on the basis of their position. For phenological and growth traits, IC 64 and IC 65 were associated with flowering and maturity stability, while AFg4 and IC 69 were identified as winning genotypes for plant height and branches plant^-1^, respectively. For yield-attributing and yield traits, AFg5 and AFg4 emerged as the winning genotypes for pods plant^-1^, pod length, seed yield, and biological yield, particularly in productive environments E8 and E9, whereas HM showed specific adaptation for seeds pod^-1^. IC 65 and IC 69 were associated with test weight, whereas IC 69 emerged as the winning genotype for harvest index.

**Figure 6:**
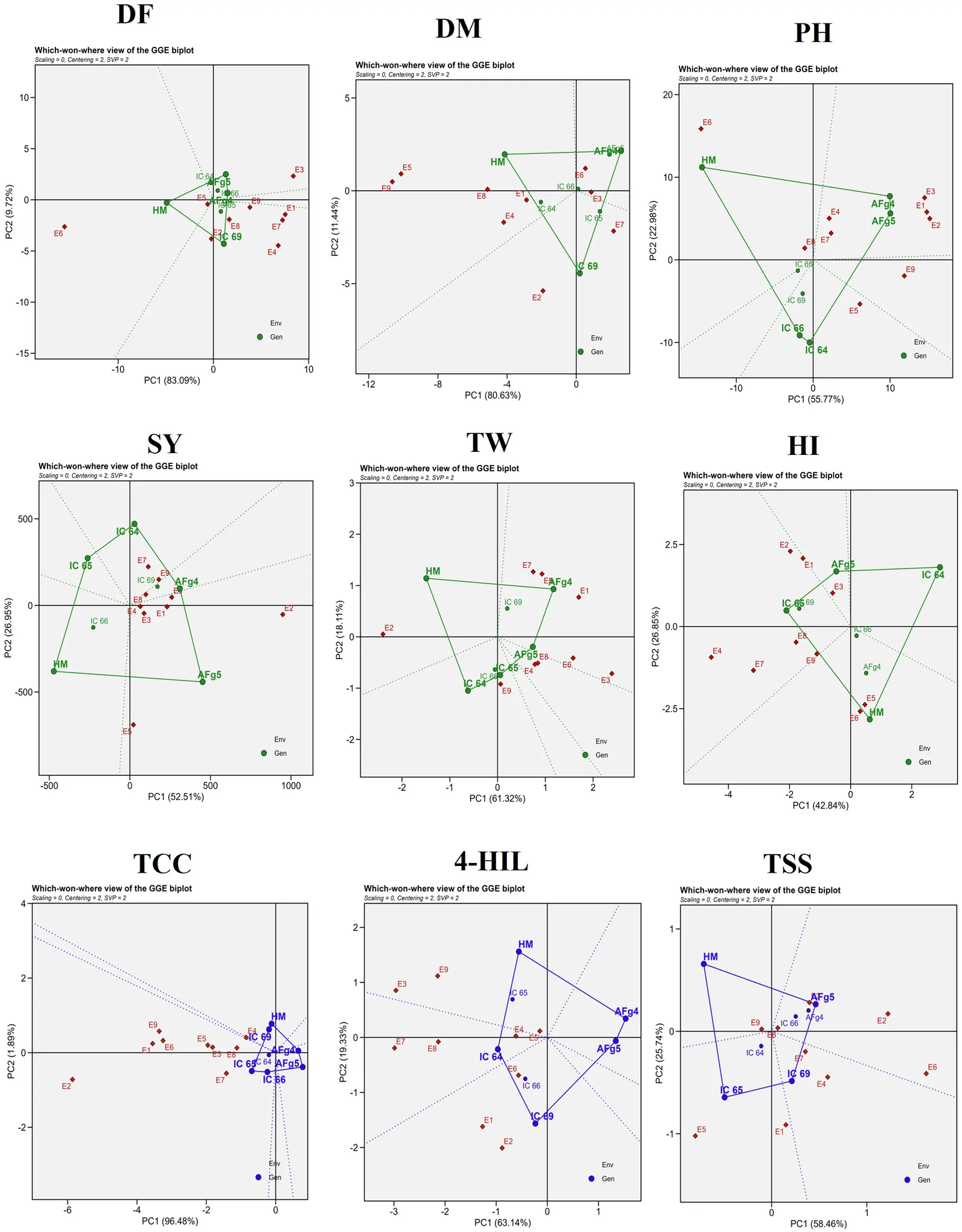
“Which-won-where” GGE biplot polygon views illustrating genotype main effects (G) and genotype × environment interactions (G × E) among seven fenugreek genotypes evaluated across nine environments for phenological, growth, and yield-attributing and biochemical traits. The polygon biplots identify superior and specifically adapted genotypes for individual test environments based on vertex genotypes and sector patterns.

Among biochemical traits, IC 65 was associated with total chlorophyll content, IC 64 with 4-hydroxyisoleucine (4-HIL), and Hisar Manohar with diosgenin and free amino acid content. IC 66 emerged as the winning genotype for protein and nitrogen contents, whereas IC 69 showed superior performance for total soluble sugar content.

Overall, the polygon-view GGE biplot identified AFg5, AFg4, and IC 69 as the principal genotypes for yield-related traits under favourable environments, whereas IC 64, IC 65, IC 66, and HM exhibited favourable adaptation for several biochemical and phenological traits, highlighting their potential for stable performance across diverse environments.

#### 3.3.5 Performance ranking of experimental environments

The GGE biplot environment-ranking analysis revealed clear differences among test locations in their suitability for evaluating phenological, growth, yield, and biochemical traits (**Figure 7**; **Supplementary Figure S6**). Environments located closer to the centre of the concentric circles were considered more desirable because they combined greater discriminating ability with higher representativeness.

**Figure 7:**
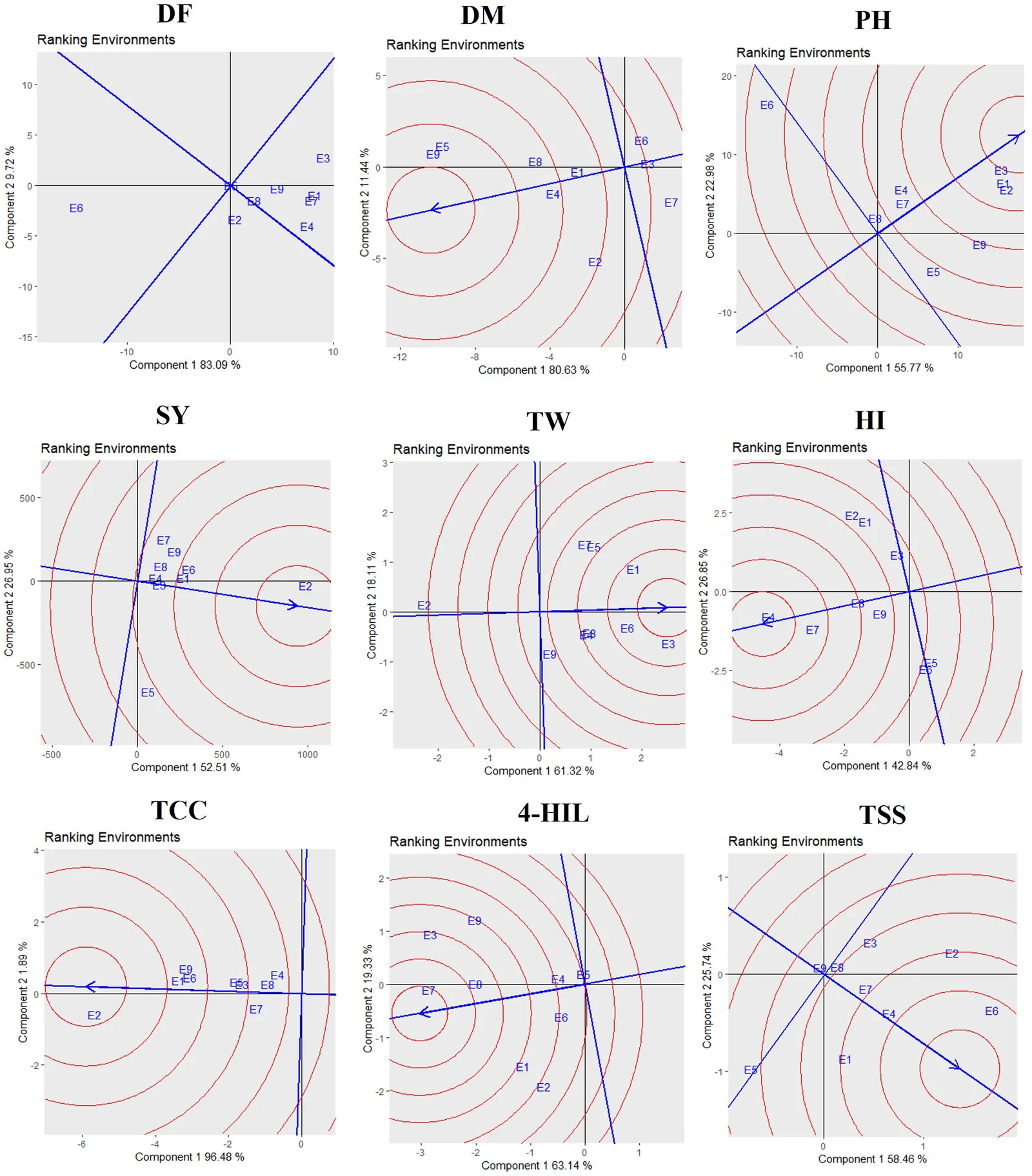
The GGE biplot “Environment ranking” pattern for environment comparison with ideal environment showing G + G × E main effect among seven fenugreek genotypes evaluated across nine environments for key phenological, growth, and yield-attributing and biochemical traits. The biplots were generated using the first two interaction principal component axes (IPCA1 and IPCA2) derived from AMMI analysis, enabling simultaneous assessment of genotype stability, environmental influence, and specific adaptation. Genotypes and environments located near the origin indicate broad adaptability and greater stability, whereas those positioned farther from the origin exhibit stronger interaction effects and specific environmental responsiveness.

For phenological traits, E1 and E5 were positioned closest to the ideal environment for days to flowering and maturity. Among growth traits, E9 ranked closest to the ideal environment for plant height, whereas E3 was the most desirable environment for branches plant^-1^. For yield-attributing and yield traits, E9 consistently ranked closest to the ideal environment for pods plant**^-^**^1^, pod length, seed yield, and biological yield, while E8 was particularly favourable for seeds pod^-^¹ and also ranked highly for several yield-related traits. Test weight and harvest index exhibited comparatively lower environmental differentiation.

For biochemical traits, E1 and E5 were consistently located nearest to the ideal environment for total chlorophyll, 4-hydroxyisoleucine (4-HIL), diosgenin, free amino acids, protein, nitrogen, and total soluble sugar. Overall, E9 and E8 emerged as the most suitable environments for evaluation of yield-related traits, whereas E1 and E5 were identified as ideal environments for assessment of biochemical traits across fenugreek genotypes.

#### 3.3.6 Factor analysis and selection differential

Factor analysis grouped the evaluated agronomic and biochemical traits into three major latent factors (FA1-FA3), reflecting the underlying structure of trait associations among fenugreek genotypes (**Table 4**). FA1 was primarily associated with productivity-related traits, including seed yield (0.845), pods plant^-1^ (0.824), pod length (0.794), seeds pod^-1^ (0.653), and test weight (0.505). FA2 was characterized mainly by 4-hydroxyisoleucine (4-HIL; 0.904) with moderate contributions from days to maturity (0.596) and TSS content (0.503). FA3 was strongly associated with nitrogen (0.937) content.

**Table 4:** Factor loadings, heritability, selection differential, and predicted selection gain for phenological, yield, and biochemical traits of seven fenugreek genotypes.

Trait	FA1	FA2	FA3	Communality	Uniqueness	Xo	Xs	SD	SD_%	h2	SG	SG_%	Sense	Goal
SY	0.845	0.131	-0.205	0.774	0.226	1683.0	1643.4	-39.6	-2.4	61.2	-24.3	-1.4	+	100
4-HIL	-0.183	0.904	0.046	0.853	0.147	9.4	9.8	0.4	4.1	85.2	0.3	3.5	+	100
DC	0.084	0.277	-0.276	0.160	0.840	192.4	191.1	-1.2	-0.7	0.0	0.0	0.0	+	100
TSS	-0.242	0.503	0.067	0.316	0.684	2.4	2.4	0.0	0.6	56.3	0.0	0.4	+	100
TCC	0.282	0.358	-0.015	0.208	0.792	2.6	3.5	0.9	33.8	95.2	0.8	32.2	+	100
PC	-0.263	0.113	0.937	0.960	0.040	27.2	27.9	0.7	2.4	92.6	0.6	2.2	+	100
FAA	-0.187	0.906	0.009	0.855	0.145	11.8	12.3	0.4	3.7	88.8	0.4	3.3	+	100
NC	-0.263	0.113	0.937	0.960	0.040	4.4	4.5	0.1	2.4	92.6	0.1	2.2	+	100
BY	0.188	0.042	-0.367	0.172	0.828	7032.5	6612.8	-419.7	-6.0	61.5	-257.9	-3.7	+	100
DM	0.142	0.596	0.138	0.394	0.606	126.2	125.6	-0.5	-0.4	75.3	-0.4	-0.3	–	0
DF	-0.125	-0.335	0.060	0.131	0.869	55.1	55.2	0.1	0.1	6.0	0.0	0.0	–	0
HI	-0.560	-0.273	0.223	0.438	0.562	25.3	25.7	0.4	1.5	68.5	0.3	1.0	+	100
BPP	0.282	0.083	0.304	0.179	0.821	6.2	6.3	0.1	1.8	45.9	0.1	0.8	+	100
PH	0.452	-0.408	-0.112	0.383	0.617	71.3	70.1	-1.3	-1.8	63.3	-0.8	-1.1	–	0
PL	0.794	-0.250	-0.271	0.767	0.233	10.4	10.2	-0.1	-1.3	2.9	0.0	0.0	+	100
PPP	0.824	-0.111	-0.193	0.728	0.272	48.4	47.3	-1.1	-2.3	14.1	-0.2	-0.3	+	100
SPP	0.653	-0.223	-0.191	0.513	0.487	17.3	17.4	0.2	0.8	54.1	0.1	0.5	+	100
TW	0.505	0.108	-0.019	0.267	0.733	12.8	12.6	-0.2	-1.9	55.7	-0.1	-1.0	+	100

FA, Factor analysis; Xo, Mean of genotype; Xs, Mean of selected genotype; h2, Broad sense heritability; SD ,Selection differential; SG, Selection gain; SY,Seed yield; 4-HIL, 4-hydroxyisoleucine; DC, Diosgenin content; TSS, Total soluble sugars; TCC, Total chlorophyll content; NC, Nitrogen content; BY, Biological yield; DM, Days to maturity; DF, Days to flowering; HI, Harvest index; BPP, Branches plant^-1^; PH, Plant height; PL, Pod length; PPP, Pods plant^-1^; SPP, Seeds pod^-1^; TW, Test weight. Sense, “+” indicates that higher values are desirable, “-−” indicates that lower values are desirable

Communality estimates indicated that nitrogen (0.960), and 4-HIL (0.853) were highly explained by the extracted factors, whereas seed yield (0.774) and pod length (0.767) were moderately represented. In contrast, diosgenin (0.160), flowering days (0.131), and biological yield (0.172) exhibited comparatively low communalities, suggesting that these traits were less effectively captured by the retained factor structure.

Selection differential and predicted selection gain analyses revealed favourable gains for several target traits, particularly total chlorophyll content (32.2%), followed by 4-HIL (3.5%), FAA (3.3%), nitrogen (2.2%), harvest index (1.0%), and seeds pod^-1^ (0.5%). Conversely, negative gains were observed for days to maturity (-0.3%), plant height (-1.1%), seed yield (-1.4%), and biological yield (-3.7%). The substantial positive gains observed for chlorophyll and key biochemical traits highlight their importance in genotype discrimination and selection, particularly for improvement of green-seeded fenugreek. Overall, the extracted factor structure effectively integrated agronomic and biochemical information and provided the foundation for subsequent MTSI and MGIDI analysis.

#### 3.3.7 Multi trait stability index

The Multi-Trait Stability Index (MTSI) effectively discriminated fenugreek genotypes based on their combined performance and stability across nine contrasting environments (**Figure 8A**). As lower MTSI values indicate closer proximity to the ideotype, genotypes with lower scores are considered more desirable owing to their ability to simultaneously express favourable agronomic, yield, and biochemical traits with greater environmental stability.

**Figure 8:**
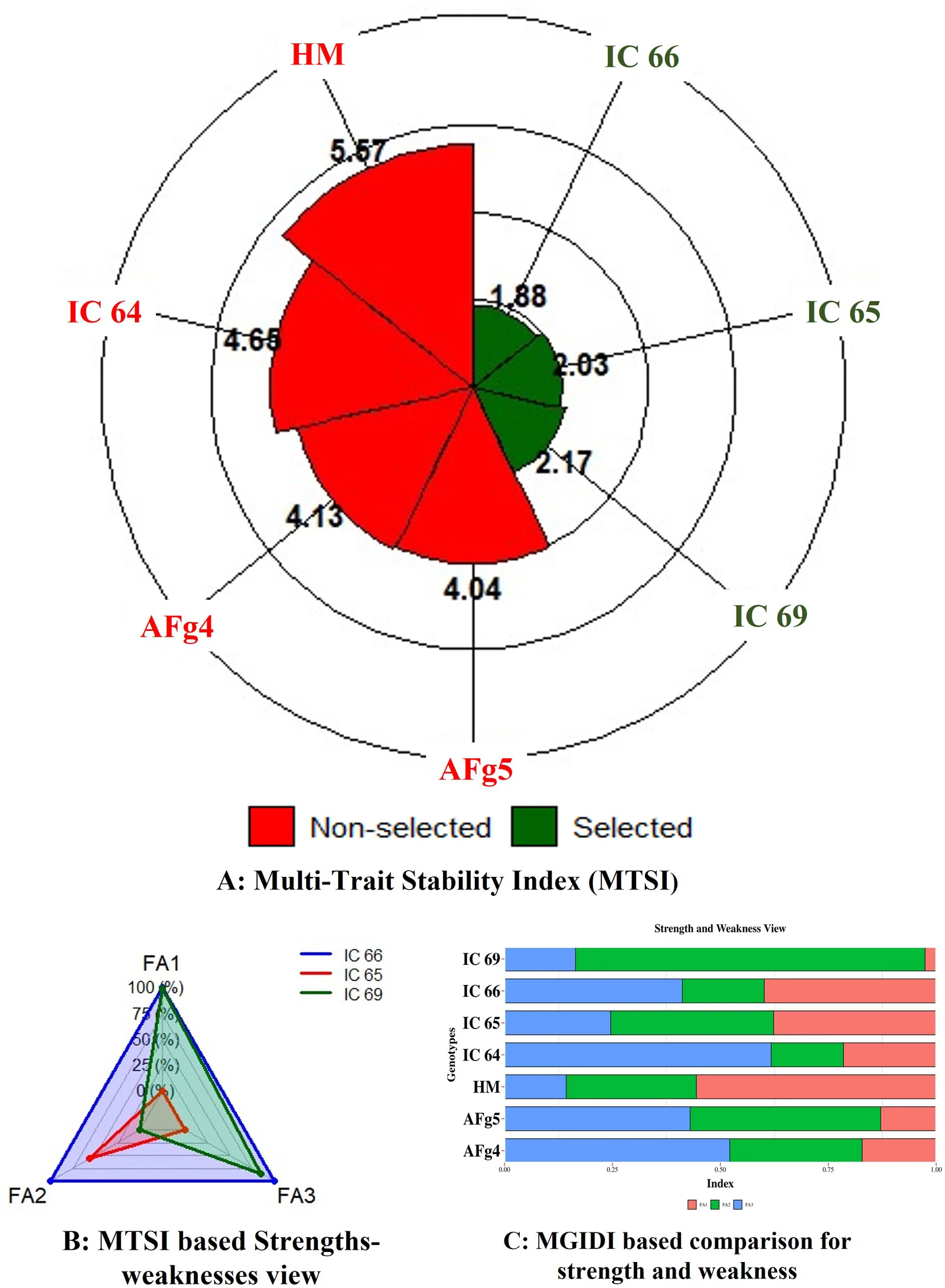
Multi-trait selection analysis of fenugreek genotypes across environments. **(A)** Multi-Trait Stability Index (MTSI) ranking based on overall performance and stability. **(B)** MTSI-based strength-weakness view of the selected genotypes showing the contribution of factor groups (FA1–FA3) to multi-trait performance. **(C)** MGIDI-based strength-weakness analysis showing the relative contribution of factor groups to genotype-ideotype distance.

Among the evaluated genotypes, the green-seeded entries IC 66 (1.88), IC 65 (2.03), and IC 69 (2.17) recorded the lowest MTSI values, indicating superior multi-trait performance, broad adaptation, and high stability across environments. In contrast, AFg5 (4.04), AFg4 (4.13), IC 64 (4.65), and HM (5.57) exhibited comparatively higher MTSI values, reflecting greater deviation from the ideotype under multi-environment conditions.

The superior ranking of IC 66, IC 65, and IC 69 demonstrates their ability to combine stable agronomic performance with favourable biochemical attributes, making them promising genetic resources for breeding fenugreek cultivars with improved yield stability and biochemical quality across diverse environments.

#### 3.3.8 Strengths and weaknesses of selected genotypes

The strengths–weaknesses analysis provided complementary information on the contribution of the underlying factor groups to the performance of the selected genotypes. The MTSI-based strengths–weaknesses view (**Figure 8B**) revealed distinct contributions of the three latent factors (FA1-FA3) to the overall performance of IC 66, IC 65, and IC 69. IC 66 exhibited a relatively balanced contribution across the three factors, whereas IC 69 showed stronger contributions from FA1 and FA3, and IC 65 was more strongly associated with FA2-related traits. These factor-specific contributions indicated that the selected genotypes differed in the trait groups contributing to their overall multi-trait performance.

The MGIDI-based strengths-weaknesses analysis (**Figure 8C**) further illustrated the relative contribution of the factor groups to the genotype–ideotype distance. The profiles demonstrated that the selected genotypes differed in their strengths and weaknesses across the underlying factor groups, providing additional information on the specific trait combinations contributing to their multivariate performance. Thus, the MGIDI analysis complemented the MTSI-based evaluation by providing an additional ideotype-oriented perspective on multi-trait selection.

#### 3.3.9 Comparative identification of stable and superior genotypes across traits

Comparative evaluation using AMMI1, AMMI2, GGE biplot, which-won-where, MTSI and MGIDI analyses identified distinct genotype responses across phenological, growth, yield, and biochemical traits (**Supplementary Tables S23**). IC 64, IC 65, and AFg4 were consistently associated with relatively stable responses for phenological traits, whereas AFg4 and IC 69 showed superior performance for growth-related traits. For yield and yield-attributing traits, AFg5, AFg4, and IC69 were repeatedly identified as superior genotypes across environments, with AFg5 showing particular superiority for seed yield and biological yield. For biochemical traits, IC 64, IC 65, IC 66, IC 69, and HM were identified as desirable genotypes for total chlorophyll, 4-HIL, diosgenin, phenol, nitrogen, and total soluble sugar content The complementary MGIDI analysis further provided an ideotype-oriented assessment of the strengths and weaknesses of the evaluated genotypes (**Figure 8C**). Based on multi-trait evaluation, MTSI ranked IC 66, IC 65, and IC 69 as the most desirable genotypes. The environment-ranking analyses further identified E9 and E8 as the most informative environments for yield-related traits, whereas E1 and E5 were most suitable for evaluation of biochemical traits.

## 4 Discussion

Identifying genotypes that combine high productivity, nutritional superiority, and wide adaptation across diverse environments is a primary objective of modern crop improvement programmes. In fenugreek, the realization of this objective is often constrained by substantial genotype × environment (G×E) interactions, which alter the expression of agronomic and biochemical traits across locations and seasons. The highly significant effects of genotype, environment, and G×E interaction observed in the present study confirm the differential response of fenugreek genotypes to varying agro-climatic conditions and highlight the necessity of multi-environment testing for reliable genotype evaluation. The use of complementary analytical approaches, including AMMI, GGE biplot, factor analysis, and MTSI and MGIDI, enabled a comprehensive assessment of performance, stability, and adaptability, thereby facilitating the identification of superior green-seeded genotypes with potential for cultivation across a wide range of production environments.

The present investigation demonstrated the existence of substantial genotype × environment (G × E) interactions for yield, yield-attributing, and biochemical traits in green-seeded fenugreek across diverse agro-climatic conditions. The significant variation among environments and differential genotype responses observed in pooled ANOVA, AMMI, and GGE analyses indicated that environmental factors played a decisive role in the expression of productivity and nutritional traits (Shahriari et al., 2018). Such crossover interactions generally reduce the efficiency of direct selection because superior genotypes in one environment may not maintain comparable performance under contrasting conditions. Therefore, identification of stable and broadly adapted genotypes using multivariate stability models becomes highly important for fenugreek improvement under multi-location testing conditions. Similar observations regarding the strong influence of environmental variability on yield and quality traits have also been reported in fenugreek and other crops grown under heterogeneous environments. The AMMI ANOVA further demonstrated that environmental effects were the predominant source of variation for most traits, particularly seed yield, biological yield, pod length, and seeds pod^-^¹, whereas genotypic effects contributed more strongly to total chlorophyll, phenol, and nitrogen contents. This indicates that productivity-related traits were largely influenced by environmental conditions, while variation in several biochemical traits was comparatively more associated with genetic differences among genotypes. The relatively high contribution of G×E for days to flowering, total soluble sugars, phenol, test weight, and plant height further indicates differential genotype responses to environmental conditions. The significant G×E interaction observed for 16 of the 17 traits confirms that genotype performance was not uniform across environments, supporting the need for multi-environment selection (Gauch et al., 2008; Olivoto and Lúcio, 2020). IPCA1 captured a major proportion of the G×E interaction for most traits, while IPCA2 accounted for additional interaction variation, with the two components together capturing 63.54-96.44% of G×E variation. The partitioning of G×E through interaction principal component axes is a central feature of AMMI analysis and facilitates interpretation of genotype-specific environmental responses (Gauch, 2006; Pour-Aboughadareh et al., 2022). These results demonstrate that the first two AMMI interaction axes adequately described the major patterns of genotype-specific environmental responsiveness and provide a quantitative basis for subsequent stability and adaptability analyses (Olivoto and Lúcio, 2020).

The substantial variation observed for SY, BY, PPP, and PL across testing environments indicated the strong influence of environmental factors on fenugreek productivity. The superior performance recorded at E9 and E8 suggested that these environments provided favourable agro-climatic conditions, including optimum temperature, better soil fertility, and suitable moisture availability, which likely enhanced assimilate production and partitioning towards reproductive sinks. In contrast, the lower productivity at E7 reflected the adverse impact of environmental stress on reproductive efficiency and biomass accumulation. Similar environmental effects on fenugreek productivity have been reported by Yadav et al. (2017), who demonstrated that environmental indices significantly affected the expression of yield-related traits across diverse sowing environments. Likewise, Kole and Saha (2013) reported considerable variation in heritability estimates for SY under different environments, highlighting the dynamic nature of genotype × environment interactions in fenugreek.

The superior performance of AFg5 and AFg4 for yield and related traits further confirmed the presence of substantial exploitable genetic variability among green-seeded fenugreek genotypes. AFg5 recorded the highest pooled SY and BY, indicating greater physiological efficiency, stronger sink strength, and enhanced adaptability across environments. Similarly, AFg4 exhibited maximum plant height and test weight, reflecting vigorous vegetative growth and improved seed development potential. These findings are consistent with the observations of Panwar et al. (2018), who reported high genotypic coefficients of variation for seed yield and associated traits in fenugreek. Furthermore, Yadav et al. (2017) observed high heritability and genetic advance for seed yield and pods per plant, suggesting the predominance of additive gene action and the effectiveness of selection for yield improvement.

A major outcome of the present study was the identification of green-seeded fenugreek genotypes that combined desirable agronomic performance with enhanced biochemical composition. Previous studies have demonstrated that green-seeded fenugreek possesses higher chlorophyll concentrations and elevated levels of bioactive compounds such as 4-HIL, FAA, and P constituents compared with conventional yellow-seeded types (Singh et al., 2025a, Singh et al., 2025b). The present multi-environment evaluation further demonstrated that these biochemical advantages were maintained across diverse agro-climatic conditions, indicating a strong genetic basis and environmental resilience of these traits. This finding is particularly important because it suggests that improvement of biochemical quality can be achieved without compromising agronomic adaptation and productivity.

AMMI analysis revealed clear differences in genotype responses across environments. Genotypes with relatively lower IPCA scores exhibited lower G × E interaction, whereas genotypes with larger scores showed greater environmental responsiveness. In the present study, IC 69 and IC 65 showed comparatively consistent performance across environments, whereas AFg5 combined high productivity with greater responsiveness to favourable environments. These contrasting responses highlight the importance of considering both mean performance and G × E interaction when identifying stable and specifically adapted genotypes. Similar effectiveness of AMMI analysis for identifying stable and widely adapted genotypes has been reported in fenugreek, chickpea, wheat, and passion fruit (Yadav et al., 2020; Balapure et al., 2016; Regmi et al., 2021; Oliveira et al., 2014; Shafii and Price, 1998).

AMMI2 analysis further indicated comparatively consistent responses of IC 69 and IC 65 across the tested environments, whereas AFg5 and AFg4 showed greater responsiveness to favourable environments. The distinct clustering of genotypes and environments in the biplot reflected substantial genotype × environment interaction, with E9, E8, and E1 contributing most strongly to genotype discrimination. These findings underscore the importance of multi-environment testing for identifying both widely adapted and specifically adapted genotypes with superior performance. Similar applications of AMMI2 in maize, rice, and other field crops have demonstrated its effectiveness in capturing interaction patterns and facilitating the identification of stable genotypes across diverse environments (Nayak et al., 2008; Babić et al., 2011; Gauch et al., 2008). Furthermore, studies in cereals and legumes have shown that integrating multiple stability statistics provides a more robust assessment of genotype performance than reliance on a single analytical approach (Bomma et al., 2024; Al-Ghumaiz et al., 2025).

The combined application of GGE biplot, genotype ranking, environment ranking, and discriminativeness-representativeness analyses revealed clear differences in genotype adaptation across environments. AFg5 consistently emerged as the superior genotype for SY, BY, and PPP under favourable environments, whereas AFg4 exhibited superiority for PH and TW. HM showed specific adaptation for SPP and diosgenin accumulation and also exhibited higher free amino acid content. In contrast, IC 66, IC 65, and IC 69 showed comparatively consistent performance across the tested environments, suggesting lower sensitivity to environmental fluctuations for several evaluated traits. Environment-ranking analyses further identified E9 and E8 as the most suitable environments for evaluation of yield-related traits, whereas E1 and E5 were more effective for assessment of biochemical attributes. Similar effectiveness of GGE biplot analysis for identifying superior genotypes and delineating mega-environments has been reported in maize, barley, chickpea, cassava, sorghum and red onion (Dehghani et al., 2006; Farshadfar et al., 2013; Aruna et al., 2016; Daemo et al., 2023; Phuke et al., 2017; Gupta et al., 2025).

The biochemical traits exhibited pronounced genotype × environment interactions; however, several green-seeded genotypes consistently maintained superior biochemical profiles across environments. IC 65 recorded the highest TCC, confirming the distinctive green-seed phenotype, whereas IC 66 exhibited superior nitrogen contents. IC 64 was distinguished by enhanced 4-HIL accumulation, while HM showed superiority for DC. The consistent expression of these traits across environments suggests a strong genetic basis for biochemical superiority in green-seeded fenugreek and highlights their potential as valuable genetic resources for the development of biochemically enriched cultivars (Kona et al., 2024).

Factor analysis (**Table 4**) provided additional insight into the relationships among agronomic and biochemical traits and served as the basis for the Multi-Trait Stability Index (MTSI) approach. The extracted factors differentiated productivity-related and biochemical traits, indicating that these trait groups contributed differently to genotype discrimination. Selection-gain analysis showed favourable gains for several biochemical traits, particularly total chlorophyll content (32.2%) and 4-HIL (3.5%), whereas seed yield (-1.4%) and biological yield (-3.7%) showed negative predicted gains. This contrasting response indicates that improvement in biochemical attributes may not necessarily be accompanied by simultaneous gains in productivity, highlighting the need to consider potential trade-offs among yield and quality traits during selection (Olivoto et al., 2019; Zuffo et al., 2020; Sampaio et al., 2023).

The Multi-Trait Stability Index (MTSI), a robust multivariate approach that simultaneously integrates productivity, stability, and trait desirability, effectively differentiated the fenugreek genotypes based on their overall performance across environments. The lowest MTSI values were recorded for IC 66 (1.88), IC 65 (2.03), and IC 69 (2.17), indicating their closer proximity to the ideotype and superior multi-trait performance (Olivoto et al., 2019). These genotypes exhibited broad adaptation and stable expression of agronomic, yield, and biochemical traits across diverse agro-climatic conditions, highlighting their potential utility in varietal development and breeding programmes. In particular, IC 69 combined stable productivity with desirable quality attributes, including higher TSS, HI, whereas IC 66 demonstrated superior performance for NC together with high overall stability. The ability of these genotypes to maintain consistent performance across environments underscores their value as genetic resources for developing climate-resilient and nutritionally enhanced fenugreek cultivars. Similar effectiveness of the MTSI approach in identifying superior and stable genotypes has been reported in taro (Chauhan et al., 2025), cassava (Koundinya et al., 2021), and bitter gourd (Moharana et al., 2026). From a breeding perspective, the MTSI results were particularly important because they identified green-seeded genotypes capable of simultaneously expressing desirable agronomic and biochemical traits with high environmental stability. The consistent selection of IC 66, IC 65, and IC 69 across multiple stability approaches suggests that these genotypes represent valuable parental resources for developing high-yielding and biochemically enriched fenugreek cultivars adapted to diverse production environments.

The MGIDI-based strengths-weaknesses analysis provided a complementary perspective on the multi-trait performance of the evaluated genotypes (**Figure 8C**). Unlike MTSI, which is specifically designed to integrate multi-trait performance with environmental stability in multi-environment trials, MGIDI evaluates the distance of genotypes from a predefined ideotype while accounting for the correlation structure among traits (Olivoto and Nardino, 2021). The MGIDI profiles further illustrated differences among genotypes in the factor groups contributing to their overall genotype-ideotype distance, providing additional insight into the trait combinations underlying their multivariate performance. Thus, MGIDI and MTSI represent complementary rather than interchangeable selection frameworks. In the present study, MTSI was retained as the principal selection criterion because environmental stability across nine contrasting environments was a central objective of genotype selection, whereas MGIDI provided an additional ideotype-oriented assessment of multi-trait performance.

The comparative assessment of AMMI, GGE biplot, MTSI and MGIDI analyses further revealed complementary patterns of genotype adaptation and stability. AFg5, AFg4, and IC 69 consistently emerged as superior genotypes for yield and yield-attributing traits, particularly under favourable environments, whereas IC 64, IC 65, and IC 66 showed comparatively consistent performance for phenological and nutritional traits across the tested environments. HM showed specific adaptation for SPP and DC accumulation, indicating its potential value as a donor parent for quality improvement. The discriminativeness versus representativeness analysis identified E9, E1, E5, and E8 as highly informative test environments, emphasizing their importance for efficient genotype evaluation and selection in future fenugreek breeding programmes.

Collectively, these analyses demonstrated the potential of green-seeded fenugreek germplasm for simultaneous improvement of productivity, biochemical quality, and environmental stability. The complementary use of stability, multi-trait, and environment-discrimination approaches provided a robust basis for identifying promising genotypes and informative test environments, supporting their utilization in breeding programmes aimed at developing high-yielding, biochemically enriched, and environmentally resilient fenugreek cultivars.

## 5 Conclusion

This study represents one of the first comprehensive multi-environment evaluations of green-seeded fenugreek germplasm for agronomic performance, stability, and biochemical attributes across diverse agro-climatic conditions. Significant genotype × environment interactions observed for most phenological, growth, yield, and biochemical traits highlighted the importance of multi-location testing for accurate identification of stable and superior genotypes. The combined application of AMMI, GGE biplot, Multi-Trait Stability Index (MTSI) and Multi-Trait Genotype-Ideotype Distance Index (MGIDI) analyses provided a robust framework for simultaneously assessing productivity, adaptability, stability, and biochemical performance across environments.

Although the check cultivar AFg5 exhibited superior performance for seed yield, biological yield, and pods plant^-1^ under favourable environments, the primary objective of the study was to evaluate the performance and stability of green-seeded fenugreek genotypes. In this regard, the green-seeded lines demonstrated remarkable adaptability and consistency across locations. Among them, IC 66, IC 65, and IC 69 emerged as the most desirable genotypes based on MTSI, MGIDI and other statistical analysis, indicating their superior multi-trait stability and broad adaptation under diverse environmental conditions. Furthermore, the green-seeded genotypes exhibited distinct biochemical advantages, with IC 65 recording higher chlorophyll content, IC 66 exhibiting superior protein and nitrogen levels, and IC 69 combining stable productivity with desirable biochemical attributes. These findings confirm that the green-seeded germplasm possesses substantial potential for simultaneously improving agronomic performance and biochemical composition.

The environmental evaluation further identified E9 and E8 as highly favourable and discriminating environments for selection of high-yielding genotypes, whereas E1 and E5 were particularly suitable for the evaluation of biochemical traits. Collectively, the findings demonstrate that green-seeded fenugreek represents a valuable genetic resource for the development of climate-resilient, stable, and value-added cultivars with enhanced biochemical attributes. The identified environments and trait-specific strengths provide useful guidance for targeted breeding and future multi-environment evaluation of promising green-seeded material.

## Data Availability

The original contributions presented in the study are included in the article/**Supplementary Material**. Further inquiries can be directed to the corresponding authors.
