# Supplementary material for: Yield stability and GxE interaction of green-seeded fenugreek across diverse environments using multivariate models
Source: Front Plant Sci. 2026 Sep 22;17:1917204. doi: 10.3389/fpls.2026.1917204 (PMC13638621; doi:10.3389/fpls.2026.1917204)
Supplement: Supplementary file 1 [file Table1.docx]

**TABLE S1:** Name of the Environment (centres) involved and Latitude, Longitude, altitude, of the experimental site

| **S. No.** | **Environment** | **ID** | **Latitude (°N)** | **Longitude (°E)** | **Altitude**  **(m AMSL)** |
| --- | --- | --- | --- | --- | --- |
| 1 | ICAR-NRCSS Ajmer | E1 | 26.368313 | 74.592815 | 324 |
| 2 | KVK Jhunjhunu | E2 | 28.101046 | 75.347351 | 450 |
| 3 | KVK Maulasar | E3 | 27.311263 | 74.754449 | 302 |
| 4 | COH, Mandsaur | E4 | 24.057024 | 75.101363 | 460 |
| 5 | ICAR-CIAH Bikaner | E5 | 28.114973 | 73.345740 | 240 |
| 6 | SKNAU Jobner | E6 | 26.977251 | 75.373267 | 400 |
| 7 | KVK Fatehpur | E7 | 28.013495 | 74.960107 | 324 |
| 8 | KVK Pratapgarh | E8 | 24.024305 | 74.815162 | 515 |
| 9 | ATC Ajmer | E9 | 26.367947 | 74.594142 | 324 |

**TABLE S2:** Rabi Season Climate Parameters (October-March) during the growing period of 2023-24 & 2024-25

| **S. No.** | **Environment** | **2023–2024** | | | | | **2024–2025** | | | | | |
| --- | --- | --- | --- | --- | --- | --- | --- | --- | --- | --- | --- | --- |
|  |  | **Rainfall (mm)** | **Temp (°C)** | | **RH (%)** | | **Rainfall (mm)** | **Temp (°C)** | | | **RH (%)** | |
|  |  |  | **Min** | **Max** | **Min** | **Max** |  | **Min** | **Max** | **Min** | | **Max** |
| 1 | E1 | 36.0 | 11.8 | 27.6 | 59.7 | 87.3 | 37.8 | 16.4 | 30.8 | 55.9 | | 84.3 |
| 2 | E2 | 15.7 | 10.4 | 28.7 | 38.4 | 85.7 | 13.7 | 10.2 | 29.4 | 40.7 | | 85.7 |
| 3 | E3 | 12.0 | 9.0 | 26.7 | 40.5 | 75.2 | 14.7 | 10.1 | 28.7 | 42.8 | | 80.7 |
| 4 | E4 | 0.22 | 13.6 | 29.2 | 45.2 | 72.1 | 14.0 | 11.9 | 29.6 | 42.5 | | 73.2 |
| 5 | E5 | 1.4 | 9.0 | 26.0 | 37.0 | 77.0 | 5.7 | 9.0 | 28.0 | 54.0 | | 78.0 |
| 6 | E6 | 21.0 | 3.3 | 38.0 | 36.1 | 69.7 | 22.9 | 4.5 | 35.4 | 36.0 | | 69.7 |
| 7 | E7 | 40.3 | 8.6 | 34.4 | 20.1 | 95.1 | 28.2 | 8.2 | 36.7 | 43.8 | | 89.6 |
| 8 | E8 | 28.7 | 10.7 | 32.7 | 35.7 | 85.1 | 30.2 | 10.2 | 33.7 | 35.7 | | 87.1 |
| 9 | E9 | 35.0 | 11.9 | 27.7 | 59.9 | 87.5 | 38.2 | 16.5 | 30.9 | 56.1 | | 85.1 |

**TABLE S3:** Basic Soil & Water Properties of Experimental Locations (Environment)

| **S. No.** | **Environment** | **OC (%)** | **Available N (kg/ha)** | **Available P (kg/ha)** | **Available K (kg/ha)** | **Soil pH** | **Soil EC (dS/m)** | **Water pH** | **Water EC (dS/m)** |
| --- | --- | --- | --- | --- | --- | --- | --- | --- | --- |
| 1 | E1 | 0.19 | 240.8 | 18.7 | 280.7 | 7.8 | 0.28 | 7.9 | 1.20 |
| 2 | E2 | 0.16 | 138.9 | 15.7 | 168.7 | 8.2 | 0.38 | 7.8 | 1.26 |
| 3 | E3 | 0.24 | 145.0 | 13.6 | 172.2 | 8.5 | 0.45 | 8.1 | 2.42 |
| 4 | E4 | 0.41 | 185.1 | 16.6 | 296.5 | 7.5 | 0.31 | 7.6 | 0.76 |
| 5 | E5 | 0.14 | 212.6 | 20.5 | 225.6 | 8.4 | 0.14 | 7.6 | 0.84 |
| 6 | E6 | 0.15 | 133.6 | 20.4 | 217.8 | 8.3 | 0.51 | 7.8 | 1.00 |
| 7 | E7 | 0.12 | 119.3 | 17.1 | 260.5 | 9.3 | 0.14 | 8.1 | 1.40 |
| 8 | E8 | 0.37 | 225.7 | 30.5 | 280.7 | 7.7 | 0.27 | 7.6 | 0.87 |
| 9 | E9 | 0.21 | 265.2 | 24.0 | 155.0 | 8.1 | 0.13 | 7.8 | 3.04 |

**TABLE S4.** Mean Performance of Fenugreek Genotypes for Plant Height (cm) Across Different Environment Along with ANOVA Parameters

| Environment  /Genotype | IC64 | IC65 | IC66 | IC69 | AFg4 | AFg5 | HM | Mean Environment |
| --- | --- | --- | --- | --- | --- | --- | --- | --- |
| E1 | 72.0 | 73.9 | 71.0 | 76.7 | 84.2 | 85.8 | 68.5 | 76.0 |
| E2 | 71.9 | 70.4 | 66.7 | 66.6 | 82.4 | 82.1 | 67.0 | 72.4 |
| E3 | 61.0 | 67.4 | 68.3 | 77.4 | 77.3 | 65.8 | 69.3 | 69.5 |
| E4 | 78.5 | 79.1 | 85.1 | 82.1 | 82.0 | 80.2 | 72.3 | 79.9 |
| E5 | 63.1 | 71.8 | 66.7 | 66.8 | 69.6 | 68.7 | 90.1 | 70.9 |
| E6 | 62.8 | 67.2 | 71.4 | 76.6 | 70.8 | 74.2 | 70.0 | 70.4 |
| E7 | 48.5 | 49.1 | 48.4 | 48.5 | 49.0 | 48.9 | 50.6 | 49.0 |
| E8 | 77.8 | 77.8 | 81.5 | 78.3 | 84.5 | 84.2 | 69.6 | 79.1 |
| E9 | 73.9 | 74.8 | 71.0 | 68.6 | 83.9 | 83.9 | 67.0 | 74.7 |
| Mean Genotype | 67.7 | 70.1 | 70.0 | 71.3 | 75.9 | 74.8 | 69.3 |  |
| Factors | | C.D. | SE(d) | SE(m) |  |  |  |  |
| Factor (E, Environment) | | 3.3 | 1.7 | 1.2 |  |  |  |  |
| Factor (G, Genotype) | | 2.9 | 1.5 | 1.0 |  |  |  |  |
| Factor (E X G) | | 8.6 | 4.4 | 3.1 |  |  |  |  |

**TABLE S5.** Mean Performance of Fenugreek Genotypes for Number of Branches per Plant Across Different Environment Along with ANOVA Parameters

| Environment  /Genotype | IC64 | IC65 | | IC66 | IC69 | AFg4 | AFg5 | HM | Mean Environment |
| --- | --- | --- | --- | --- | --- | --- | --- | --- | --- |
| E1 | 6.05 | 7.00 | | 6.43 | 6.45 | 5.98 | 5.98 | 6.23 | 6.30 |
| E2 | 6.23 | 6.55 | | 6.28 | 6.38 | 6.08 | 6.05 | 6.30 | 6.26 |
| E3 | 7.53 | 7.35 | | 6.90 | 7.43 | 6.65 | 7.00 | 8.75 | 7.37 |
| E4 | 5.25 | 5.43 | | 5.60 | 6.10 | 5.58 | 5.43 | 5.28 | 5.52 |
| E5 | 6.35 | 6.85 | | 6.28 | 7.23 | 6.68 | 6.50 | 6.35 | 6.60 |
| E6 | 5.38 | 6.25 | | 5.55 | 5.43 | 5.55 | 6.28 | 5.50 | 5.70 |
| E7 | 4.75 | 4.63 | | 5.38 | 5.38 | 5.50 | 5.50 | 4.25 | 5.05 |
| E8 | 5.98 | 6.25 | | 6.20 | 6.43 | 6.15 | 5.68 | 5.80 | 6.07 |
| E9 | 6.40 | 7.15 | | 6.80 | 7.05 | 6.40 | 5.85 | 6.30 | 6.56 |
| Mean Genotype | 5.99 | 6.38 | | 6.16 | 6.43 | 6.06 | 6.03 | 6.08 |  |
| Factors | | | C.D. | SE(d) | SE(m) |  |  |  |  |
| Factor (E, Environment) | | | 0.34 | 0.17 | 0.12 |  |  |  |  |
| Factor (G, Genotype) | | | 0.30 | 0.15 | 0.11 |  |  |  |  |
| Factor (E X G) | | | 0.90 | 0.46 | 0.32 |  |  |  |  |

**TABLE S6.** Mean Performance of Fenugreek Genotypes for Number of Pods per Plant Across Different Environment Along with ANOVA Parameters

| Environment  /Genotype | IC64 | IC65 | IC66 | IC69 | AFg4 | AFg5 | HM | Mean Environment |
| --- | --- | --- | --- | --- | --- | --- | --- | --- |
| E1 | 53.8 | 56.7 | 52.5 | 60.9 | 60.1 | 61.3 | 61.8 | 58.1 |
| E2 | 41.8 | 41.2 | 42.0 | 39.9 | 44.5 | 42.1 | 43.8 | 42.2 |
| E3 | 44.8 | 50.0 | 51.5 | 56.5 | 43.6 | 56.5 | 49.8 | 50.4 |
| E4 | 41.4 | 37.3 | 46.6 | 35.6 | 47.3 | 40.0 | 63.5 | 44.5 |
| E5 | 31.8 | 30.2 | 30.7 | 26.3 | 33.1 | 35.2 | 43.5 | 33.0 |
| E6 | 38.3 | 36.9 | 34.3 | 35.7 | 31.5 | 38.7 | 40.8 | 36.6 |
| E7 | 37.1 | 36.4 | 38.4 | 34.5 | 27.1 | 34.3 | 23.5 | 33.0 |
| E8 | 62.5 | 61.3 | 66.9 | 49.9 | 76.1 | 71.2 | 63.6 | 64.5 |
| E9 | 68.5 | 68.1 | 70.6 | 76.1 | 86.2 | 82.0 | 63.8 | 73.6 |
| Mean Genotype | 46.7 | 46.4 | 48.2 | 46.2 | 49.9 | 51.2 | 50.4 |  |
| Factors | | C.D. | SE(d) | SE(m) |  |  |  |  |
| Factor (E, Environment) | | 3.1 | 1.6 | 1.1 |  |  |  |  |
| Factor (G, Genotype) | | 2.7 | 1.4 | 1.0 |  |  |  |  |
| Factor (E X G) | | 8.1 | 4.1 | 2.9 |  |  |  |  |

**TABLE S7.** Mean Performance of Fenugreek Genotypes for Pod Length (cm) Across Different Environment Along with ANOVA Parameters

| Environment  /Genotype | IC64 | IC65 | IC66 | IC69 | AFg4 | AFg5 | HM | Mean Environment |
| --- | --- | --- | --- | --- | --- | --- | --- | --- |
| E1 | 11.13 | 11.05 | 11.18 | 10.68 | 11.20 | 11.50 | 11.60 | 11.19 |
| E2 | 10.93 | 10.73 | 10.63 | 9.75 | 10.90 | 11.33 | 11.88 | 10.88 |
| E3 | 9.00 | 9.03 | 9.43 | 9.63 | 9.78 | 9.03 | 9.00 | 9.27 |
| E4 | 11.63 | 10.40 | 10.53 | 11.40 | 11.25 | 11.05 | 10.25 | 10.93 |
| E5 | 8.88 | 8.78 | 7.88 | 8.38 | 8.88 | 7.98 | 9.00 | 8.54 |
| E6 | 10.10 | 9.83 | 9.70 | 9.25 | 9.80 | 10.35 | 10.08 | 9.87 |
| E7 | 8.23 | 8.28 | 8.65 | 8.35 | 8.18 | 8.63 | 9.10 | 8.49 |
| E8 | 12.43 | 11.60 | 11.70 | 11.25 | 11.88 | 12.40 | 11.08 | 11.76 |
| E9 | 12.50 | 12.95 | 11.88 | 12.95 | 12.63 | 12.68 | 11.18 | 12.39 |
| Mean Genotype | 10.53 | 10.29 | 10.17 | 10.18 | 10.50 | 10.55 | 10.35 |  |
| Factors | | C.D. | SE(d) | SE(m) |  |  |  |  |
| Factor (E, Environment) | | 0.32 | 0.16 | 0.11 |  |  |  |  |
| Factor (G, Genotype) | | 0.28 | 0.14 | 0.10 |  |  |  |  |
| Factor (E X G) | | 0.83 | 0.42 | 0.30 |  |  |  |  |

**TABLE S8.** Mean Performance of Fenugreek Genotypes for Number of Seeds per Pod Across Different Environment Along with ANOVA Parameters

| Environment  /Genotype | IC64 | IC65 | IC66 | IC69 | AFg4 | AFg5 | HM | Mean Environment |
| --- | --- | --- | --- | --- | --- | --- | --- | --- |
| E1 | 17.33 | 18.10 | 17.65 | 18.33 | 17.55 | 17.48 | 19.55 | 18.00 |
| E2 | 17.43 | 17.45 | 17.65 | 16.80 | 17.20 | 17.80 | 19.80 | 17.73 |
| E3 | 16.88 | 17.50 | 17.63 | 16.75 | 16.13 | 15.75 | 15.50 | 16.59 |
| E4 | 18.55 | 18.38 | 18.93 | 19.65 | 18.48 | 18.10 | 19.08 | 18.74 |
| E5 | 16.00 | 16.63 | 16.38 | 15.80 | 15.73 | 15.43 | 16.28 | 16.03 |
| E6 | 15.53 | 16.23 | 15.15 | 14.95 | 15.78 | 15.95 | 15.30 | 15.55 |
| E7 | 14.63 | 13.75 | 14.63 | 14.25 | 13.88 | 14.13 | 15.00 | 14.32 |
| E8 | 18.93 | 18.95 | 19.75 | 20.10 | 19.25 | 18.98 | 19.45 | 19.34 |
| E9 | 19.30 | 19.45 | 19.80 | 19.45 | 18.65 | 19.25 | 19.80 | 19.39 |
| Mean Genotype | 17.17 | 17.38 | 17.51 | 17.34 | 16.96 | 16.98 | 17.75 |  |
| Factors | | C.D. | SE(d) | SE(m) |  |  |  |  |
| Factor (E, Environment) | | 0.622 | 0.315 | 0.223 |  |  |  |  |
| Factor (G, Genotype) | | NS | 0.278 | 0.196 |  |  |  |  |
| Factor (E X G) | | NS | 0.833 | 0.589 |  |  |  |  |

NS: non-significant

**TABLE S9.** Mean Performance of Fenugreek Genotypes for Days to 50% Flowering Across Different Environment Along with ANOVA Parameters

| Environment  /Genotype | IC64 | IC65 | IC66 | IC69 | AFg4 | AFg5 | HM | Mean Environment |
| --- | --- | --- | --- | --- | --- | --- | --- | --- |
| E1 | 50.4 | 50.1 | 50.0 | 51.3 | 49.5 | 49.0 | 49.5 | 50.0 |
| E2 | 51.5 | 53.3 | 52.3 | 53.4 | 50.6 | 50.3 | 49.0 | 51.5 |
| E3 | 51.1 | 51.1 | 51.5 | 51.1 | 51.4 | 48.9 | 48.8 | 50.6 |
| E4 | 53.5 | 52.6 | 55.4 | 53.7 | 56.6 | 52.3 | 57.6 | 54.5 |
| E5 | 60.9 | 58.0 | 57.5 | 59.0 | 56.3 | 53.8 | 70.0 | 59.3 |
| E6 | 54.8 | 55.9 | 54.8 | 55.6 | 54.9 | 52.6 | 53.0 | 54.5 |
| E7 | 50.9 | 52.0 | 50.6 | 55.1 | 52.8 | 51.8 | 50.0 | 51.9 |
| E8 | 54.5 | 56.8 | 59.3 | 57.0 | 59.8 | 54.9 | 53.3 | 56.5 |
| E9 | 49.0 | 50.5 | 49.0 | 52.0 | 47.0 | 47.0 | 49.0 | 49.1 |
| Mean Genotype | 52.9 | 53.4 | 53.4 | 54.2 | 53.2 | 51.2 | 53.4 |  |
| Factors | | C.D. | SE(d) | SE(m) |  |  |  |  |
| Factor (E, Environment) | | 0.47 | 0.24 | 0.17 |  |  |  |  |
| Factor (G, Genotype) | | 0.41 | 0.21 | 0.15 |  |  |  |  |
| Factor (E X G) | | 1.24 | 0.63 | 0.45 |  |  |  |  |

**TABLE S10.** Mean Performance of Fenugreek Genotypes for Test Weight (g) Across Different Environment Along with ANOVA Parameters

| Environment  /Genotype | IC64 | IC65 | IC66 | IC69 | AFg4 | AFg5 | HM | Mean Environment |
| --- | --- | --- | --- | --- | --- | --- | --- | --- |
| E1 | 13.60 | 13.60 | 14.03 | 14.60 | 15.50 | 15.15 | 13.53 | 14.29 |
| E2 | 12.43 | 12.15 | 12.23 | 12.20 | 13.43 | 13.48 | 10.25 | 12.31 |
| E3 | 12.00 | 11.70 | 12.38 | 11.85 | 12.03 | 12.58 | 11.15 | 11.95 |
| E4 | 10.75 | 10.98 | 10.78 | 11.53 | 12.65 | 11.75 | 11.50 | 11.42 |
| E5 | 12.10 | 12.23 | 11.75 | 12.60 | 12.75 | 12.28 | 10.50 | 12.03 |
| E6 | 13.70 | 13.88 | 13.35 | 15.10 | 15.23 | 13.93 | 14.13 | 14.19 |
| E7 | 11.60 | 11.70 | 11.75 | 11.55 | 11.80 | 12.00 | 10.73 | 11.59 |
| E8 | 13.83 | 13.63 | 12.50 | 12.90 | 12.85 | 12.65 | 12.28 | 12.95 |
| E9 | 16.15 | 13.48 | 13.93 | 14.48 | 13.35 | 13.95 | 13.03 | 14.05 |
| Mean Genotype | 12.91 | 12.59 | 12.52 | 12.98 | 13.29 | 13.08 | 11.90 |  |
| Factors | | C.D. | SE(d) | SE(m) |  |  |  |  |
| Factor (E, Environment) | | 0.22 | 0.11 | 0.08 |  |  |  |  |
| Factor (G, Genotype) | | 0.19 | 0.10 | 0.07 |  |  |  |  |
| Factor (E X G) | | 0.58 | 0.29 | 0.21 |  |  |  |  |

**TABLE S11.** Mean Performance of Fenugreek Genotypes for Seed Yield (kg ha⁻¹) Across Different Environment Along with ANOVA Parameters

| Environment  /Genotype | IC64 | IC65 | IC66 | IC69 | AFg4 | AFg5 | HM | Mean Environment |
| --- | --- | --- | --- | --- | --- | --- | --- | --- |
| E1 | 2,351 | 2,300 | 2,290 | 2,395 | 2,457 | 2,492 | 2,228 | 2,359 |
| E2 | 1,258 | 1,183 | 1,115 | 1,152 | 1,187 | 1,340 | 1,208 | 1,206 |
| E3 | 843 | 1,073 | 896 | 969 | 1,064 | 998 | 916 | 966 |
| E4 | 725 | 1,017 | 1,323 | 1,169 | 1,081 | 1,578 | 1,448 | 1,192 |
| E5 | 1,695 | 1,562 | 1,708 | 1,635 | 1,841 | 1,756 | 1,443 | 1,663 |
| E6 | 985 | 1,249 | 799 | 1,169 | 1,136 | 961 | 873 | 1,025 |
| E7 | 921 | 969 | 982 | 994 | 903 | 971 | 755 | 928 |
| E8 | 2,931 | 2,844 | 3,042 | 3,063 | 2,716 | 2,917 | 2,507 | 2,860 |
| E9 | 2,990 | 2,597 | 2,635 | 3,132 | 3,326 | 3,493 | 2,483 | 2,951 |
| Mean Genotype | 1,633 | 1,644 | 1,643 | 1,742 | 1,746 | 1,834 | 1,540 |  |
| Factors | | C.D. | SE(d) | SE(m) |  |  |  |  |
| Factor (E, Environment) | | 102.2 | 51.7 | 36.6 |  |  |  |  |
| Factor (G, Genotype) | | 90.1 | 45.6 | 32.3 |  |  |  |  |
| Factor (E X G) | | 270.3 | 136.9 | 96.8 |  |  |  |  |

**TABLE S12**. Mean Performance of Fenugreek Genotypes for Biological Yield (kg ha⁻¹) Across Different Environment Along with ANOVA Parameters

| Environment  /Genotype | IC64 | IC65 | IC66 | IC69 | AFg4 | AFg5 | HM | Mean Environment |
| --- | --- | --- | --- | --- | --- | --- | --- | --- |
| E1 | 11,143 | 10,388 | 10,704 | 10,764 | 12,698 | 11,589 | 11,291 | 11,225 |
| E2 | 5,080 | 4,691 | 4,505 | 4,589 | 5,067 | 5,207 | 4,972 | 4,873 |
| E3 | 3,842 | 3,827 | 3,282 | 3,659 | 3,936 | 3,590 | 3,478 | 3,659 |
| E4 | 2,568 | 3,600 | 4,623 | 4,004 | 3,715 | 5,448 | 4,755 | 4,102 |
| E5 | 6,171 | 5,598 | 5,936 | 5,850 | 6,581 | 6,688 | 4,694 | 5,931 |
| E6 | 3,996 | 4,411 | 3,022 | 4,025 | 4,172 | 3,843 | 3,261 | 3,819 |
| E7 | 3,724 | 3,724 | 4,047 | 3,735 | 3,799 | 3,999 | 2,828 | 3,694 |
| E8 | 12,079 | 10,886 | 11,597 | 12,037 | 10,736 | 11,518 | 9,643 | 11,214 |
| E9 | 16,881 | 12,123 | 12,070 | 16,315 | 17,177 | 17,316 | 11,562 | 14,778 |
| Mean Genotype | 7,276 | 6,583 | 6,643 | 7,220 | 7,542 | 7,688 | 6,276 |  |
| Factors | | C.D. | SE(d) | SE(m) |  |  |  |  |
| Factor (E, Environment) | | 480 | 243 | 172 |  |  |  |  |
| Factor (G, Genotype) | | 423 | 214 | 152 |  |  |  |  |
| Factor (E X G) | | 1270 | 643 | 455 |  |  |  |  |

**TABLE S13.** Mean Performance of Fenugreek Genotypes for Harvest Index (%) Across Different Environment Along with ANOVA Parameters

| Environment  /Genotype | IC64 | IC65 | IC66 | IC69 | AFg4 | AFg5 | HM | Mean Environment |
| --- | --- | --- | --- | --- | --- | --- | --- | --- |
| E1 | 21.24 | 22.41 | 21.75 | 22.44 | 19.65 | 21.79 | 19.74 | 21.29 |
| E2 | 24.73 | 25.20 | 24.58 | 24.86 | 23.60 | 25.66 | 24.49 | 24.73 |
| E3 | 22.40 | 28.86 | 27.23 | 27.30 | 27.17 | 28.01 | 26.28 | 26.75 |
| E4 | 27.03 | 27.10 | 27.21 | 27.15 | 27.38 | 27.24 | 30.43 | 27.65 |
| E5 | 28.12 | 28.38 | 29.67 | 28.70 | 28.30 | 27.11 | 31.17 | 28.78 |
| E6 | 24.47 | 28.47 | 26.37 | 28.99 | 28.24 | 26.01 | 26.80 | 27.05 |
| E7 | 25.00 | 26.74 | 24.51 | 27.57 | 23.92 | 24.54 | 26.80 | 25.58 |
| E8 | 24.29 | 26.17 | 26.27 | 25.49 | 25.31 | 25.42 | 26.02 | 25.57 |
| E9 | 20.24 | 21.60 | 19.58 | 21.91 | 17.91 | 21.56 | 19.49 | 20.32 |
| Mean Genotype | 24.17 | 26.10 | 25.24 | 26.05 | 24.61 | 25.26 | 25.69 |  |
| Factors | | C.D. | SE(d) | SE(m) |  |  |  |  |
| Factor (E, Environment) | | 1.01 | 0.51 | 0.36 |  |  |  |  |
| Factor (G, Genotype) | | 0.89 | 0.45 | 0.32 |  |  |  |  |
| Factor (E X G) | | 2.68 | 1.36 | 0.96 |  |  |  |  |

**TABLE S14.** Mean Performance of Fenugreek Genotypes for Days to Maturity Across Different Environment Along with ANOVA Parameters

| Environment  /Genotype | IC64 | IC65 | IC66 | IC69 | AFg4 | AFg5 | HM | Mean Environment |
| --- | --- | --- | --- | --- | --- | --- | --- | --- |
| E1 | 128.8 | 128.9 | 128.4 | 130.4 | 128.0 | 128.4 | 132.3 | 129.3 |
| E2 | 126.2 | 126.3 | 126.6 | 126.4 | 126.5 | 126.8 | 125.5 | 126.3 |
| E3 | 125.5 | 121.6 | 122.5 | 122.9 | 119.4 | 120.8 | 124.0 | 122.4 |
| E4 | 114.4 | 108.0 | 110.9 | 109.5 | 106.0 | 108.2 | 118.1 | 110.7 |
| E5 | 132.8 | 131.7 | 132.4 | 133.0 | 134.7 | 133.7 | 133.3 | 133.1 |
| E6 | 129.7 | 129.3 | 128.5 | 129.6 | 128.9 | 128.2 | 125.3 | 128.5 |
| E7 | 129.1 | 128.2 | 127.1 | 128.7 | 127.0 | 126.3 | 133.3 | 128.5 |
| E8 | 132.5 | 125.0 | 128.3 | 127.0 | 123.8 | 124.3 | 135.3 | 128.0 |
| E9 | 129.0 | 129.0 | 128.5 | 133.3 | 126.0 | 126.3 | 128.8 | 128.7 |
| Mean Genotype | 127.6 | 125.3 | 125.9 | 126.7 | 124.5 | 124.7 | 128.4 |  |
| Factors | | C.D. | SE(d) | SE(m) |  |  |  |  |
| Factor (E, Environment) | | 0.62 | 0.32 | 0.22 |  |  |  |  |
| Factor (G, Genotype) | | 0.55 | 0.28 | 0.20 |  |  |  |  |
| Factor (E X G) | | 1.65 | 0.83 | 0.59 |  |  |  |  |

**TABLE S15.** Mean Performance of Fenugreek Genotypes for Seed Nitrogen Content (%) Across Different Locations Along with ANOVA Parameters

| Environment  /Genotype | IC64 | IC65 | IC66 | IC69 | AFg4 | AFg5 | HM | Mean Environment |
| --- | --- | --- | --- | --- | --- | --- | --- | --- |
| E1 | 3.89 | 4.03 | 4.07 | 4.13 | 4.03 | 4.04 | 4.03 | 4.03 |
| E2 | 4.29 | 4.32 | 4.88 | 4.50 | 4.49 | 4.49 | 4.03 | 4.43 |
| E3 | 4.94 | 4.90 | 4.82 | 4.83 | 4.63 | 4.66 | 4.12 | 4.70 |
| E4 | 4.26 | 3.98 | 4.19 | 4.37 | 4.22 | 3.96 | 3.79 | 4.11 |
| E5 | 4.80 | 4.94 | 4.88 | 4.81 | 4.62 | 4.65 | 4.05 | 4.68 |
| E6 | 4.69 | 4.67 | 4.67 | 4.79 | 4.62 | 4.57 | 4.08 | 4.58 |
| E7 | 4.68 | 4.59 | 4.54 | 4.60 | 4.36 | 4.28 | 4.18 | 4.46 |
| E8 | 4.23 | 4.19 | 4.13 | 4.10 | 4.07 | 4.03 | 3.96 | 4.10 |
| E9 | 4.22 | 4.20 | 4.40 | 4.32 | 4.13 | 3.86 | 3.89 | 4.14 |
| Mean Genotype | 4.44 | 4.42 | 4.51 | 4.49 | 4.35 | 4.28 | 4.01 |  |
| Factors | | C.D. | SE(d) | SE(m) |  |  |  |  |
| Factor (E, Environment) | | 0.12 | 0.06 | 0.04 |  |  |  |  |
| Factor (G, Genotype) | | 0.11 | 0.06 | 0.04 |  |  |  |  |
| Factor (E X G) | | 0.33 | 0.17 | 0.12 |  |  |  |  |

**TABLE S 16.** Mean Performance of Fenugreek Genotypes for Seed Protein Content (%) Across Different Locations Along with ANOVA Parameters

| Environment  /Genotype | IC64 | IC65 | IC66 | IC69 | AFg4 | AFg5 | HM | Mean Environment |
| --- | --- | --- | --- | --- | --- | --- | --- | --- |
| E1 | 24.32 | 25.21 | 25.43 | 25.82 | 25.17 | 25.23 | 25.18 | 25.19 |
| E2 | 26.80 | 26.99 | 30.50 | 28.13 | 28.08 | 28.05 | 25.17 | 27.67 |
| E3 | 30.82 | 30.64 | 30.13 | 30.19 | 28.93 | 29.10 | 25.78 | 29.37 |
| E4 | 26.62 | 24.85 | 26.16 | 27.31 | 26.38 | 24.75 | 23.68 | 25.68 |
| E5 | 30.01 | 30.87 | 30.47 | 30.04 | 28.90 | 29.04 | 25.31 | 29.23 |
| E6 | 29.32 | 29.16 | 29.17 | 29.94 | 28.87 | 28.54 | 25.52 | 28.64 |
| E7 | 29.26 | 28.65 | 28.35 | 28.77 | 27.23 | 26.77 | 26.12 | 27.88 |
| E8 | 26.44 | 26.15 | 25.80 | 25.60 | 25.41 | 25.16 | 24.75 | 25.62 |
| E9 | 26.40 | 26.22 | 27.48 | 26.96 | 25.80 | 24.09 | 24.29 | 25.89 |
| Mean Genotype | 27.78 | 27.64 | 28.16 | 28.08 | 27.19 | 26.75 | 25.09 |  |
| Factors | | C.D. | SE(d) | SE(m) |  |  |  |  |
| Factor (E, Environment) | | 0.77 | 0.39 | 0.28 |  |  |  |  |
| Factor (G, Genotype) | | 0.68 | 0.35 | 0.24 |  |  |  |  |
| Factor (E X G) | | 2.05 | 1.04 | 0.73 |  |  |  |  |

**TABLE S 17.** Mean Performance of Fenugreek Genotypes for Total Chlorophyll Content in Seed (mg g⁻¹ seed) Across Different Environment Along with ANOVA Parameters

| Environment  /Genotype | IC64 | IC65 | IC66 | IC69 | AFg4 | AFg5 | HM | Mean Environment |
| --- | --- | --- | --- | --- | --- | --- | --- | --- |
| E1 | 3.67 | 5.27 | 3.63 | 3.93 | 1.50 | 1.11 | 3.74 | 3.26 |
| E2 | 2.82 | 2.92 | 2.58 | 2.65 | 1.25 | 1.02 | 2.31 | 2.22 |
| E3 | 2.02 | 2.14 | 1.92 | 2.21 | 1.46 | 1.23 | 2.20 | 1.88 |
| E4 | 2.61 | 3.18 | 2.60 | 2.73 | 1.22 | 1.06 | 2.53 | 2.27 |
| E5 | 4.65 | 5.28 | 4.33 | 4.30 | 2.30 | 1.59 | 4.30 | 3.82 |
| E6 | 1.83 | 2.13 | 1.77 | 1.69 | 0.61 | 0.54 | 0.62 | 1.31 |
| E7 | 2.15 | 2.41 | 2.15 | 2.23 | 1.31 | 1.25 | 2.07 | 1.94 |
| E8 | 3.05 | 4.47 | 3.22 | 3.78 | 1.12 | 0.66 | 3.37 | 2.81 |
| E9 | 4.69 | 7.43 | 5.36 | 4.27 | 0.92 | 0.66 | 4.29 | 3.95 |
| Mean Genotype | 3.05 | 3.91 | 3.06 | 3.09 | 1.30 | 1.01 | 2.83 |  |
| Factors | | C.D. | SE(d) | SE(m) |  |  |  |  |
| Factor (E, Environment) | | 0.29 | 0.14 | 0.10 |  |  |  |  |
| Factor (G, Genotype) | | 0.25 | 0.13 | 0.09 |  |  |  |  |
| Factor (E X G) | | 0.75 | 0.38 | 0.27 |  |  |  |  |

**TABLE S18.** Mean Performance of Fenugreek Genotypes for Diosgenin Content (mg 100 g⁻¹ seed) Across Different Environment Along with ANOVA Parameters

| Environment  /Genotype | IC64 | IC65 | IC66 | IC69 | AFg4 | AFg5 | HM | Mean Environment |
| --- | --- | --- | --- | --- | --- | --- | --- | --- |
| E1 | 321.0 | 342.1 | 289.5 | 320.5 | 355.8 | 251.6 | 354.2 | 319.2 |
| E2 | 172.0 | 155.9 | 191.5 | 201.4 | 141.4 | 253.9 | 208.8 | 189.3 |
| E3 | 142.8 | 147.2 | 159.8 | 199.2 | 131.9 | 179.4 | 122.2 | 154.6 |
| E4 | 178.3 | 183.4 | 192.6 | 232.5 | 197.5 | 235.7 | 232.8 | 207.5 |
| E5 | 178.6 | 175.1 | 198.5 | 167.0 | 193.0 | 214.8 | 194.4 | 188.8 |
| E6 | 172.2 | 204.4 | 196.1 | 191.2 | 179.7 | 187.1 | 171.6 | 186.0 |
| E7 | 162.4 | 162.6 | 168.6 | 173.4 | 212.0 | 143.2 | 191.9 | 173.4 |
| E8 | 113.1 | 167.7 | 168.7 | 159.7 | 150.3 | 137.4 | 170.9 | 152.5 |
| E9 | 159.1 | 174.5 | 162.4 | 157.0 | 159.3 | 130.9 | 176.2 | 159.9 |
| Mean Genotype | 177.7 | 190.3 | 192.0 | 200.2 | 191.2 | 192.7 | 202.5 |  |
| Factors | | C.D. | SE(d) | SE(m) |  |  |  |  |
| Factor (E, Environment) | | 7.4 | 3.7 | 2.6 |  |  |  |  |
| Factor (G, Genotype) | | 6.5 | 3.3 | 2.3 |  |  |  |  |
| Factor (E X G) | | 19.5 | 9.9 | 7.0 |  |  |  |  |

**TABLE S19.** Mean Performance of Fenugreek Genotypes for Total Sugar Content (mg g⁻¹) Across Different Environment Along with ANOVA Parameters

| Environment  /Genotype | IC64 | IC65 | IC66 | IC69 | AFg4 | AFg5 | HM | Mean Environment |
| --- | --- | --- | --- | --- | --- | --- | --- | --- |
| E1 | 3.20 | 3.25 | 2.59 | 2.65 | 2.64 | 2.59 | 1.85 | 2.68 |
| E2 | 2.09 | 1.82 | 1.97 | 1.82 | 2.50 | 2.31 | 1.91 | 2.06 |
| E3 | 2.32 | 2.37 | 2.54 | 2.85 | 2.50 | 2.50 | 1.82 | 2.41 |
| E4 | 2.05 | 3.06 | 1.68 | 2.56 | 1.69 | 1.53 | 2.09 | 2.10 |
| E5 | 3.25 | 2.72 | 3.83 | 4.17 | 3.75 | 3.81 | 2.20 | 3.39 |
| E6 | 1.25 | 1.60 | 1.30 | 1.52 | 1.65 | 1.78 | 1.11 | 1.46 |
| E7 | 3.19 | 3.07 | 3.09 | 3.14 | 3.19 | 3.18 | 3.12 | 3.14 |
| E8 | 1.96 | 1.62 | 2.01 | 1.80 | 1.58 | 1.52 | 1.83 | 1.76 |
| E9 | 2.05 | 1.81 | 2.49 | 2.26 | 2.82 | 2.98 | 1.55 | 2.28 |
| Mean Genotype | 2.37 | 2.37 | 2.39 | 2.53 | 2.48 | 2.47 | 1.94 |  |
| Factors | | C.D. | SE(d) | SE(m) |  |  |  |  |
| Factor (E, Environment) | | 0.10 | 0.05 | 0.04 |  |  |  |  |
| Factor (G, Genotype) | | 0.09 | 0.04 | 0.03 |  |  |  |  |
| Factor (E X G) | | 0.26 | 0.13 | 0.09 |  |  |  |  |

**TABLE S20.** Mean Performance of Fenugreek Genotypes for Total Phenol Content (mg g⁻¹) Across Different Environment Along with ANOVA Parameters

| Environment  /Genotype | IC64 | IC65 | IC66 | IC69 | AFg4 | AFg5 | HM | Mean Environment |
| --- | --- | --- | --- | --- | --- | --- | --- | --- |
| E1 | 0.94 | 0.92 | 1.06 | 1.02 | 0.94 | 1.00 | 0.87 | 0.96 |
| E2 | 1.19 | 1.08 | 1.30 | 1.32 | 1.04 | 1.19 | 1.31 | 1.20 |
| E3 | 1.07 | 1.02 | 1.20 | 1.18 | 0.94 | 1.06 | 1.03 | 1.07 |
| E4 | 1.02 | 0.98 | 1.13 | 1.16 | 0.89 | 1.01 | 1.17 | 1.05 |
| E5 | 1.26 | 1.04 | 1.36 | 1.33 | 1.08 | 1.11 | 1.14 | 1.19 |
| E6 | 1.33 | 1.10 | 1.10 | 1.27 | 1.17 | 1.15 | 1.33 | 1.21 |
| E7 | 1.12 | 1.01 | 1.16 | 1.18 | 0.96 | 1.15 | 1.19 | 1.11 |
| E8 | 1.08 | 1.12 | 1.19 | 1.22 | 0.95 | 1.14 | 1.14 | 1.12 |
| E9 | 0.89 | 0.95 | 1.04 | 0.95 | 0.84 | 1.05 | 0.89 | 0.94 |
| Mean Genotype | 1.10 | 1.02 | 1.17 | 1.18 | 0.98 | 1.09 | 1.12 |  |
| Factors | | C.D. | SE(d) | SE(m) |  |  |  |  |
| Factor (E, Environment) | | 0.03 | 0.01 | 0.01 |  |  |  |  |
| Factor (G, Genotype) | | 0.03 | 0.01 | 0.01 |  |  |  |  |
| Factor (E X G) | | 0.07 | 0.04 | 0.03 |  |  |  |  |

**TABLE S21**. Mean Performance of Fenugreek Genotypes for Free Amino Acid (FAA) Content (mg g⁻¹) Across Different Environment Along with ANOVA Parameters

| Environment  /Genotype | IC64 | IC65 | IC66 | IC69 | AFg4 | AFg5 | HM | Mean Environment |
| --- | --- | --- | --- | --- | --- | --- | --- | --- |
| E1 | 14.96 | 14.10 | 15.35 | 14.28 | 12.89 | 13.66 | 16.58 | 14.55 |
| E2 | 14.00 | 13.29 | 13.92 | 12.54 | 11.10 | 10.26 | 14.99 | 12.87 |
| E3 | 11.61 | 11.08 | 11.32 | 10.46 | 10.49 | 10.58 | 10.81 | 10.91 |
| E4 | 9.36 | 9.28 | 9.77 | 8.99 | 9.89 | 8.43 | 9.30 | 9.29 |
| E5 | 14.92 | 14.11 | 13.68 | 15.11 | 13.06 | 14.55 | 14.57 | 14.29 |
| E6 | 12.53 | 12.03 | 10.23 | 12.08 | 8.39 | 8.91 | 11.57 | 10.82 |
| E7 | 14.04 | 14.37 | 14.34 | 13.93 | 11.75 | 12.06 | 14.51 | 13.57 |
| E8 | 10.31 | 10.92 | 9.28 | 7.60 | 7.26 | 8.08 | 9.62 | 9.01 |
| E9 | 12.00 | 10.86 | 12.98 | 9.57 | 10.59 | 10.36 | 12.33 | 11.24 |
| Mean Genotype | 12.64 | 12.23 | 12.32 | 11.62 | 10.60 | 10.76 | 12.70 |  |
| Factors | | C.D. | SE(d) | SE(m) |  |  |  |  |
| Factor (E, Environment) | | 0.42 | 0.21 | 0.15 |  |  |  |  |
| Factor (G, Genotype) | | 0.37 | 0.19 | 0.13 |  |  |  |  |
| Factor (E X G) | | 1.10 | 0.56 | 0.40 |  |  |  |  |

**TABLE S22.** Mean Performance of Fenugreek Genotypes for 4-Hydroxyisoleucine (4-HIL) Content (mg g⁻¹) Across Different Environment Along with ANOVA Parameters

| Environment  /Genotype | IC64 | IC65 | IC66 | IC69 | AFg4 | AFg5 | HM | Mean Environment |
| --- | --- | --- | --- | --- | --- | --- | --- | --- |
| E1 | 12.0 | 11.4 | 12.3 | 12.6 | 10.3 | 10.9 | 10.8 | 11.5 |
| E2 | 11.2 | 10.6 | 11.1 | 10.0 | 8.9 | 8.2 | 12.0 | 10.3 |
| E3 | 9.3 | 8.9 | 9.1 | 8.4 | 8.4 | 8.5 | 8.7 | 8.7 |
| E4 | 7.5 | 7.4 | 7.8 | 7.2 | 7.9 | 6.7 | 7.4 | 7.4 |
| E5 | 11.9 | 11.3 | 10.9 | 12.1 | 10.4 | 11.6 | 11.7 | 11.4 |
| E6 | 10.0 | 9.6 | 8.2 | 9.7 | 6.7 | 7.1 | 9.3 | 8.7 |
| E7 | 11.2 | 11.5 | 11.5 | 11.1 | 9.4 | 9.6 | 11.4 | 10.8 |
| E8 | 8.2 | 8.7 | 7.4 | 6.1 | 5.8 | 6.5 | 7.7 | 7.2 |
| E9 | 9.6 | 8.7 | 10.4 | 9.9 | 8.5 | 8.3 | 7.7 | 9.0 |
| Mean Genotype | 10.1 | 9.8 | 9.9 | 9.7 | 8.5 | 8.6 | 9.6 |  |
| Factors | | C.D. | SE(d) | SE(m) |  |  |  |  |
| Factor (E, Environment) | | 0.33 | 0.17 | 0.12 |  |  |  |  |
| Factor (G, Genotype) | | 0.29 | 0.15 | 0.10 |  |  |  |  |
| Factor (E X G) | | 0.87 | 0.44 | 0.31 |  |  |  |  |

**TABLE S23:** Comparative Identification of Stable and Superior Fenugreek Genotypes for Phenological, Yield, and Biochemical Traits Using AMMI, GGE Biplot, and MTSI Analyses Across Different Environments

| **Trait*** | **Statistical techniques** | | | | | | |
| --- | --- | --- | --- | --- | --- | --- | --- |
|  | **AMMI1** | **AMMI2** | **Which-won-where** | **GGE biplot** | **Selected genotypes** | **D/R biplot** |  |
| DF | IC64, IC65, AFg4 | IC64, IC65, AFg4 | IC64, IC65 | IC64, IC65 | IC64, IC65 | E1, E5 |  |
| DM | IC66, IC65, AFg4 | IC66, IC65, AFg4 | IC65, AFg4 | IC65, AFg4 | IC66, AFg4 | E1, E5 |  |
| PH | AFg4, IC69 | AFg4, IC69 | AFg4 | AFg4 | AFg4 | E9 |  |
| BPP | IC69, IC65 | IC69, IC65 | IC69 | IC69 | IC69 | E3 |  |
| PPP | AFg5, AFg4 | AFg5, AFg4 | AFg5, AFg4 | AFg5, AFg4 | AFg5 | E9, E8 |  |
| PL | AFg5, IC65 | AFg5, IC65 | AFg5 | AFg5 | AFg5 | E9 |  |
| SPP | HM, IC66 | HM, IC66 | HM | HM | HM | E8 |  |
| SY | AFg5, AFg4, IC69 | AFg5, AFg4, IC69 | AFg5, AFg4, IC69 | AFg5, AFg4, IC69 | AFg5, IC69 | E9, E8 |  |
| BY | AFg5, AFg4 | AFg5, AFg4 | AFg5 | AFg5 | AFg5, AFg4 | E9 |  |
| TW | IC65, IC69 | IC65, IC69 | IC65, IC69 | IC65, IC69 | IC65, IC69 | E2 |  |
| HI | IC65, IC69 | IC65, IC69 | IC69 | IC69 | IC69 | E4 |  |
| TCC | IC65, IC64 | IC65, IC64 | IC65 | IC65 | IC65 | E1, E5 |  |
| 4-HIL | IC64, IC66 | IC64, IC66 | IC64 | IC64 | IC64 | E1 |  |
| DC | HM, IC69 | HM, IC69 | HM | HM | HM | E1 |  |
| P | IC69, IC66 | IC69, IC66 | IC69, IC66 | IC69, IC66 | IC69 | E6 |  |
| NC | IC66 | IC66 | IC66 | IC66 | IC66 | E5 |  |
| TSS | IC69 | IC69 | IC69 | IC69 | IC69 | E1 |  |

*Abbreviations: Days to flowering (DF), Days to maturity (DM), Plant height (PH), Branches plant**^-1^** (BPP), Pods plant**^-1^** (PPP), Pod length (PL), Seeds pod**^-1^** (SPP), Seed yield (SY), Biological yield (BY), Test weight (TW) and Harvest index (HI), Total chlorophyll content (TCC), 4-hydroxyisoleucine (4-HIL), Diosgenin content (DC), Phenol (P), Nitrogen content (NC), Total soluble sugars (TSS), IC-0633364 (IC64), IC-0633365 (IC65), IC-0633366 (IC66), and IC-0633369 (IC69), Ajmer Fenugreek-4 (AFg4), Ajmer Fenugreek-5 (AFg5), and Hisar Manohar (HM).

**TABLE S24:** Fenugreek date of sowing (DOS) and harvesting (DOH) during 2023-2024 & 2024-2025 at all nine environments (Locations)

| **S. No.** | **Location** | **2023–2024** | | **2024–2025** | |
| --- | --- | --- | --- | --- | --- |
|  |  | **Date of Sowing** | **Days to maturity** | **Date of Sowing** | **Days to maturity** |
| 1 | ICAR-NRCSS, Tabiji, Ajmer | 01-11-2023 | 128 | 26-10-2024 | 131 |
| 2 | KVK Jhunjhunu | 09-11-2023 | 127 | 15-11-2024 | 126 |
| 3 | KVK Maulasar (Nagaur) | 20-11-2023 | 126 | 15-11-2024 | 119 |
| 4 | COH, Mandsaur (MP) | 21-11-2023 | 112 | 16-11-2024 | 109 |
| 5 | ICAR-CIAH Bikaner | 08-11-2023 | 137 | 10-11-2024 | 129 |
| 6 | SKNAU, Jobner | 11-11-2023 | 131 | 14-11-2024 | 126 |
| 7 | KVK Fatehpur (Sikar) | 06-11-2023 | 123 | 09-11-2024 | 134 |
| 8 | KVK Pratapgarh | 08-11-2023 | 128 | 11-11-2024 | 128 |
| 9 | ATC Ajmer | 06-11-2023 | 129 | 23-10-2024 | 129 |
